# Dissipation-enabled hydrodynamic conductivity in a tunable bandgap semiconductor

**DOI:** 10.1126/sciadv.abi8481

**Published:** 2022-04-15

**Authors:** Cheng Tan, Derek Y. H. Ho, Lei Wang, Jia I. A. Li, Indra Yudhistira, Daniel A. Rhodes, Takashi Taniguchi, Kenji Watanabe, Kenneth Shepard, Paul L. McEuen, Cory R. Dean, Shaffique Adam, James Hone

**Affiliations:** 1 Department of Mechanical Engineering, Columbia University, New York, NY 10027, USA.; 2 Department of Electrical Engineering, Columbia University, New York, NY 10027, USA.; 3 Yale-NUS College, 16 College Avenue West, Singapore 138614, Singapore.; 4 Centre for Advanced 2D Materials and Graphene Research Centre, National University of Singapore, 6 Science Drive 2, Singapore 117546, Singapore.; 5 Kavli Institute at Cornell for Nanoscale Science, Ithaca, NY 14853, USA.; 6 Laboratory of Atomic and Solid State Physics, Cornell University, Ithaca, NY 14853, USA.; 7 National Laboratory of Solid-State Microstructures, School of Physics, Nanjing University, Nanjing, 210093, China.; 8 Department of Physics, Brown University, Providence, RI 02912, USA.; 9 Department of Physics, National University of Singapore, 2 Science Drive 3, Singapore 117551, Singapore.; 10 National Institute for Materials Science, 1-1 Namiki, Tsukuba 305-0044, Japan.; 11 Department of Physics, Columbia University, New York, NY 10027, USA.; 12 Department of Materials Science and Engineering, National University of Singapore, 9 Engineering Drive 1, Singapore 117575, Singapore.

## Abstract

Electronic transport in the regime where carrier-carrier collisions are the dominant scattering mechanism has taken on new relevance with the advent of ultraclean two-dimensional materials. Here, we present a combined theoretical and experimental study of ambipolar hydrodynamic transport in bilayer graphene demonstrating that the conductivity is given by the sum of two Drude-like terms that describe relative motion between electrons and holes, and the collective motion of the electron-hole plasma. As predicted, the measured conductivity of gapless, charge-neutral bilayer graphene is sample- and temperature-independent over a wide range. Away from neutrality, the electron-hole conductivity collapses to a single curve, and a set of just four fitting parameters provides quantitative agreement between theory and experiment at all densities, temperatures, and gaps measured. This work validates recent theories for dissipation-enabled hydrodynamic conductivity and creates a link between semiconductor physics and the emerging field of viscous electronics.

## Teaser

Theoretical and experimental studies of electron-hole friction limited transport in bilayer graphene with a tunable bandgap.

## INTRODUCTION

More than 50 years ago, it was predicted that it was possible for electron transport to be described by macroscopic equations of motion similar to those in classical fluid mechanics (*1*, *2*). This regime is of particular relevance to low-dimensional materials such as graphene, for which interactions are intrinsically strong and disorder can be low. Within this emerging class of hydrodynamic materials, ambipolar conductors with coexisting electrons and holes (such as semimetals or small-gap semiconductors at finite temperature) are of particular interest because electron-hole scattering does not conserve current. Therefore, ambipolar materials can, in principle, act as hydrodynamic conductors in which electron-hole scattering plays a dominant role in determining the conductivity, making them a promising platform for detailed experimental and theoretical exploration of hydrodynamic behavior. Such systems are predicted (*3*–*7*) to display rich new phenomena beyond the diffusive or ballistic transport of effectively independent carriers seen in most metals, have the potential for technological application [for example, in the generation of terahertz radiation (*8*)], and are a readily accessible bridge between strongly correlated quantum fluids observed in otherwise unrelated fields such as quark-gluon plasmas in ion colliders and ultracold atomic Fermi gasses in optical traps (*9*).

A notable prediction of theory is that, at precise charge neutrality, carrier-carrier collisions occur at a quantum critical (Planckian) rate *k*_B_*T*/ħ (*10*–*12*), which can lead to temperature-independent conductivity when electron-hole scattering is dominant. Planckian dissipation has recently been measured through terahertz spectroscopy of graphene (*13*), and temperature-independent conductivity has been observed in suspended bilayer graphene (*14*). However, the former study required optical excitation of carriers to observe dominant electron-hole scattering, while the latter yielded inconsistent results across samples and the temperature range was limited to 100 K and below. Therefore, there still exists no experimental platform that shows intrinsic hydrodynamic conductivity over a wide temperature range with sufficient repeatability to validate theoretical models.

The behavior of hydrodynamic conductors away from charge neutrality is less well understood. An observed scaling of conductivity with chemical potential in suspended bilayer graphene was initially interpreted as evidence of electron-hole–limited conductivity away from neutrality (*14*). However, from a first-principles viewpoint, electron-hole scattering cannot affect the net current away from perfect neutrality. Instead, more recent theory has pointed to the importance of interaction between electron-hole scattering and momentum-nonconserving (dissipative) scattering from defects and phonons. While these mechanisms might naively be expected to add independently to electron-hole scattering as encapsulated in Matthiessen’s rule, theory instead predicts that a more complex interplay between these processes determines conductivity, in what has been described as a dissipation-enabled hydrodynamic regime (*11*, *12*). This theory has not yet been experimentally tested. Last, we note that hydrodynamic conductivity is completely unexplored (theoretically or experimentally) for gapped materials.

## RESULTS AND DISCUSSION

Here, we adopt the two-fluid formalism of (*12*), which assumes that electrons and holes each form a fluid in local equilibrium, a condition that holds in the hydrodynamic regime. The two-fluid model reproduces a numerical solution of the full quantum Boltzmann equation (*15*) and is largely in agreement with the three-mode ansatz of (*11*). For simplicity, we assume that the electron and hole bands are parabolic with the same effective mass—a standard approximation for many semimetals including bilayer graphene. While the conductivity predicted by this model is in general a complicated function of the carrier densities and relaxation times (see the Supplementary Materials), a simple picture emerges in the limit τ_0_ ≪ τ_dis_, where τ_0_ is the electron-hole relaxation time, and τ_dis_ is the relaxation time from dissipative mechanisms. We find

$$\sigma =\frac{4n_{e}n_{h}}{\left(n_{e}+n_{h}\right)}\;\frac{e^{2}}{m^{*}}\tau_{0}+\frac{{\left(n_{e}-n_{h}\right)}^{2}}{\left(n_{e}+n_{h}\right)}\;\frac{e^{2}}{m^{*}}\tau_{\text{dis}}$$ (1)

where *n_e_* and *n_h_* are the densities of thermally excited electrons and holes, and m* is the effective mass.

**Fig. 1.**
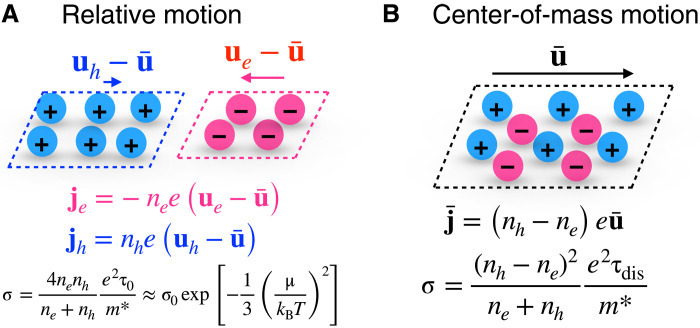
Schematic of dissipative hydrodynamics. In the limit of strong Coulomb interactions τ_0_ ≪ τ_dis_, the two-fluid model decomposes into two additive components (see Eq. 1). The relative motion between electrons and holes (**A**) is the universal Coulomb drag that dominates at charge neutrality. Away from neutrality, it decays as the number of minority carriers. The center-of-mass motion (**B**) is the nonuniversal linear response of the collective electron-hole plasma under an electric field and reduces to the usual Drude conductivity far from neutrality. Together, these two components fully describe the dissipation-enabled hydrodynamics, giving a smooth crossover from universal to nonuniversal behavior as carrier density is tuned away from neutrality.

As depicted in Fig. 1, this equation has a simple physical interpretation, in which the first and second terms describe the relative and center-of-mass motion, respectively, of the electrons and holes [we note that a similar decoupling between relative and center-of-mass motion was conjectured to explain the σ ∼ *T*^2^ dependence of the bulk conductivity of titanium disulfide as a possible signature of electron-hole–dominated scattering (*16*); however, unlike the present case, the conductivity arising from the relative motion is neither temperature independent nor universal, and comparisons between theory and experiment are complicated by imperfect sample stoichiometry disagreeing by over an order of magnitude]. The conductivity due to relative motion is limited by Coulomb drag with relaxation time τ_0_ and is maximized at charge neutrality (*n_e_* = *n_h_*). Close to charge neutrality, this term can be expressed as σ_0_ exp [−(1/3)(μ/(*k*_B_*T*))^2^], where μ is the chemical potential and *k*_B_*T* is the product of the Boltzmann constant and temperature (see eq. S14 for the more general case). Here, σ_0_ = (*e*^2^/*h*) × 8 log (2)/α_0_, where *h* is Planck’s constant and α_0_ ∼ 0.2 is a dimensionless constant that characterizes the electron-hole coupling strength (*10*–*12*). We highlight that this temperature-independent hydrodynamic conductivity σ_0_ not only is independent of the degree of disorder showing no sample-to-sample variation [similar, e.g., to mesoscopic universal conductance fluctuations (*17*)] but also is insensitive to materials parameters such as m* in the strongly interacting limit (see section S6 for detailed discussion).

The center-of-mass motion is described by a Drude model for a plasma with charge density −(*n_e_* − *n_h_*)*e*, mass density (*n_e_* + *n_h_*)m*, and momentum-nonconserving scattering time τ_dis_. This term is zero at charge neutrality and equivalent to the conventional Drude conductivity in the unipolar regime. Together, Eq. 1 captures the full crossover from the universal behavior at charge neutrality to nonuniversal behavior away from charge neutrality.

We next establish that ultraclean bilayer graphene encapsulated in hexagonal boron nitride (hBN) can act as a model system to compare theory to experiment. Graphene has emerged in recent years as an excellent platform for the study of hydrodynamics (*13*, *18*–*20*) due to its low disorder, weak electron-phonon coupling, and strong carrier-carrier interactions. Bilayer graphene has electron and hole bands that are well described by hyperbolic bands, with dispersion $\epsilon_{\pm }(k)=\pm \sqrt{{\left(\hbar^{2}k^{2}/(2m^{*})\right)}^{2}+{\left(\Delta /2\right)}^{2}}$, where ± denotes the conduction and valence bands with effective mass m* ≈ 0.03 *m_e_*, and a bandgap Δ that is tunable by an out-of-plane electric field. It is even better suited for the study of hydrodynamic conductivity than monolayer: The hydrodynamic regime is 1000× less sensitive to disorder at low temperature and should exhibit no high-temperature cutoff due to weaker coupling between electrons and optical phonons (*10*). hBN encapsulation (*21*) provides disorder approaching that of suspended graphene while suppressing flexural phonons and providing a wider range of sample geometry. Dual-gated structures offer independent tuning of carrier density and bandgap.

For this study, five dual-gated devices with Hall bar geometry and channel size from 2 to 10 μm were fabricated, all of which showed substantially identical behavior. Low-temperature conductivity and Hall effect measurements (fig. S2) were used to calibrate top and bottom capacitances, allowing calculation of μ and Δ as a function of the top and bottom gate voltages using the hyperbolic band structure (see Materials and Methods). Using this calibration, we measured the conductivity σ for gapless bilayer graphene as a function of temperature for μ = 0 and as a function of μ at a series of fixed temperatures. This was then repeated for different values of Δ.

**Fig. 2.**
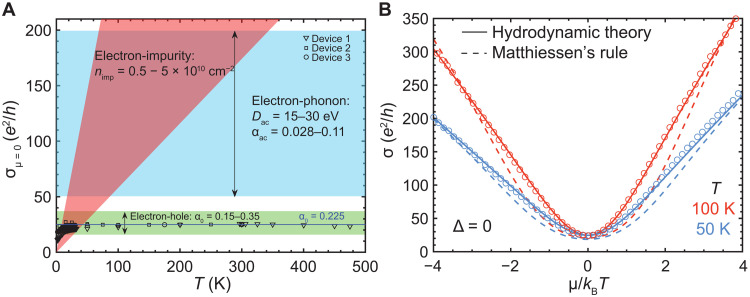
Robust hydrodynamic conductivity in bilayer graphene. (**A**) Measured charge neutral conductivity as a function of temperature for the gapless case for three devices (symbols). The green-shaded window shows the expectation from the theoretical literature for the electron-hole–limited conductivity 0.15 < α_0_ < 0.35 (*10*–*12*). Similarly, the blue region represents literature estimates of phonon-limited conductivity 0.03 < α_ac_ < 0.11 (*32*–*34*), and red for impurity-limited conductivity 0.8 ps < τ_imp_ < 8 ps, corresponding to a charged impurity density range of (0.5 − 5) × 10^10^cm^−2^ (*18*, *19*, *35*). The solid line is the best fit for the electron-hole–limited conductivity α_0_ = 0.225 ± 0.002. (**B**) For a given temperature, the experimental data (symbols) can be fit using the dissipative hydrodynamic theory (Eq. 1, solid lines) where, to account for a slight electron and hole asymmetry in the data, we allow for τ_dis_ to be different for electrons and holes. The dashed lines show a fit using a phenomenological “Matthiessen’s rule” where the resistance channels are added together in series. The disagreement with experiment shows that the momentum-conserving and momentum-nonconserving scattering do not act independently. In addition, the Matthiessen’s rule conductivity is below the experimentally observed values, which is unphysical.

Figure 2A compares experiment to literature estimates of hydrodynamic, phonon-limited, and impurity-limited conductivity for the gapless case (Δ = 0) at μ = 0. At this point, the system is charge neutral (*n_e_* = *n_h_*), and free carriers are generated solely by thermal excitation, with *n*_*e*, *h*_ ∝ *T*. The temperature-independent hydrodynamic conductivity is given by σ_0_ as discussed above (where the range in values for α_0_ in the theoretical literature does not arise from any expected variation in the experimental value but rather from the level of approximation in the calculation). The scattering time due to acoustic and substrate polar optical phonons has been calculated numerically using standard expressions available in the literature [e.g., (*22*, *23*); see section S3.2]. Unlike the case of monolayer graphene, acoustic phonon scattering is dominant over optical phonons at all temperatures and leads to scattering time of τ = (α_ac_*k*_B_*T*)^−1^ħ, where α_ac_ is the (temperature- and density-independent) bilayer graphene electron-phonon coupling strength (*10*) that varies as the square of the deformation potential *D*. The shaded region shows conductivity for the reported values of α_ac_ in the literature that correspond to *D* between 15 and 30 eV. Scattering from charged impurities was calculated using the standard expression (*24*), yielding a scattering time τ_imp_ that is nearly temperature and density independent (within 20%), leading to conductivity that increases linearly with temperature. τ_imp_ is inversely proportional to the charged impurity density *n*_imp_, which can be estimated from Hall effect measurements to fall within the range 5 × 10^9^cm^−2^ < *n*_imp_ < 5 × 10^10^ cm^−2^. See section S3 for a detailed discussion of all the relevant scattering mechanisms.

The solid points in Fig. 2A show data for three different devices. All show identical, constant conductivity with a best fit value of (24.7 ± 0.2) *e*^2^/*h* over a wide temperature range of 50 to 500 K, which falls within the range for electron-hole–limited conductivity. This finding confirms the earlier observation in suspended bilayer graphene and extends the temperature range by a factor of 5. The magnitude of the conductivity falls well outside the range for acoustic phonon scattering. Likewise, temperature-independent conductivity cannot be explained by charged impurity scattering; however, we note that the observed downturn in conductivity below 50 K is consistent with the calculated impurity-limited conductivity and that the conductivity at high density (shown below) matches predictions for acoustic phonon scattering. We thus conclude that, between 50 and 500 K, the charge-neutral conductivity is determined by electron-hole scattering, and we find experimentally that α_0_ = 0.225 ± 0.002, indicated by the solid line in the figure.

We next consider the behavior away from charge neutrality by plotting σ(μ) for two fixed temperatures (Fig. 2B). The dissipative hydrodynamic theory successfully describes the transition between the hydrodynamic regime near μ = 0 and the dissipative regime at large ∣μ∣. In contrast, combining electron-hole scattering with phonon/impurity scattering through Mattheissen’s rule underestimates the conductivity at intermediate μ, which violates Kohler’s theorem (*25*); this discrepancy becomes stronger at higher temperature. This analysis already confirms that (i) gapless bilayer graphene at μ = 0 displays sample-independent hydrodynamic conductivity limited by electron-hole scattering at the Planckian rate 1/τ_0_ = α_0_*k*_B_*T*/ħ ∼ *k*_B_*T*/ħ (*26*) over a wide temperature range up to and exceeding room temperature and (ii) its conductivity away from charge neutrality cannot be accounted for by pure electron-hole scattering or by including independent scattering from phonons/impurities.

**Fig. 3.**
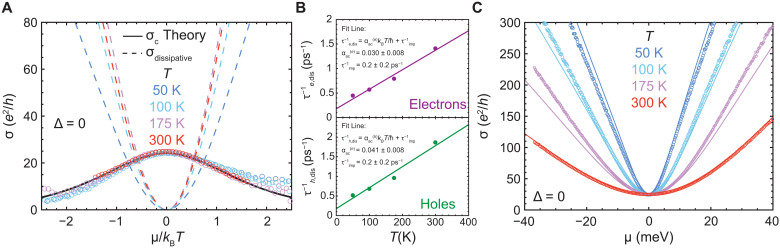
Ambipolar hydrodynamic conductivity comprises a universal and a dissipative contribution. (**A**) The decay of the universal component of the hydrodynamic conductivity away from neutrality extracted from the experiment (symbols) agrees with the theoretical calculations (solid line). The nonuniversal dissipative contribution to the hydrodynamic conductivity is also shown (dashed lines). The sum of the universal and dissipative contributions gives the solid lines in Fig. 2B. (**B**) The dissipative scattering rates $\tau_{e/h,\text{dis}}^{-1}$ extracted at different temperatures are used to obtain a single set of four global fit parameters [α_0_ = 0.225 ± 0.002 and α_ac_ = 0.030 ± 0.008 for electrons, α_ac_ = 0.041 ± 0.008 for holes, and $\tau_{\text{imp}}^{-1}=(0.2\pm 0.2)$ ps^−1^]. These four fit parameters are used in the hydrodynamic theory lines in (**C**). (C) Zero-gap conductivity measurements (symbols) as a function of μ (meV) for *T* = 50,100,175, and 300 K. The data are in excellent agreement with hydrodynamic theory developed in this work (solid lines). For the rest of this work, the same set of four global fit parameters mentioned in (B) is used consistently across the full range of carrier densities, temperature, and bandgaps.

We next extract universal Coulomb drag and dissipative contributions to the conductivity (Eq. 1) from the data. At any temperature, we can match the experimental data σ(μ) using the previously determined value α_0_ = 0.225 and two fitting parameters, τ_*e*,dis_ and τ_*h*,dis_, which represent the dissipative (phonon + impurity) scattering time for electrons and holes, respectively. The observed electron-hole asymmetry in the conductivity data is consistent with previous experiments (*27*) and necessitates fitting separately for electrons and holes. Following Eq. 1, we can obtain the dissipative component (dashed lines in Fig. 3A). This dissipative component collapses onto a single curve when acoustic phonon scattering dominates over impurity scattering, as is seen above 100 K in these devices. This collapse was previously attributed to electron-hole scattering (*14*). We next subtract the dissipative component from the total measured conductivity. As seen in the figure, the subtracted experimental data collapse onto the theoretical curve, revealing the universal behavior of electron-hole Coulomb drag scattering as a function of carrier density and temperature. At high temperature where the hydrodynamics is stronger, the agreement is excellent. To our knowledge, this universal electron-hole scattering contribution to the hydrodynamic conductivity has not been demonstrated previously, in either the theoretical or experimental literature.

The extracted values of τ_dis_(*T*) can be used to separately determine the phonon and impurity contributions to the dissipative scattering. To do so, we plot $\tau_{e/h,\text{dis}}^{-1}$ versus temperature (Fig. 3B). Because $\tau_{\text{dis}}^{-1}(T)=\alpha_{\text{ac}}k_{B}T/\hbar +\tau_{\text{imp}}^{-1}$ (see the Supplementary Materials for details), a line fit yields α_ac_ from the slope and τ_imp_ from the intercept. Following this procedure, we obtain $\alpha_{\text{ac}}^{e}=0.030\pm 0.008$, $\alpha_{\text{ac}}^{h}=0.041\pm 0.008$, and $\tau_{\text{imp}}^{-1}=0.2\pm 0.2$ ps^−1^. The derived parameters are consistent with theoretical calculations and other experimental estimates in the literature as well as other independent measurements on our samples (see section S4 for full details). The three parameters above, together with the value of α_0_ = 0.225 determined earlier, are sufficient to reproduce the entire σ(μ, *T*) dataset in the hydrodynamic regime. To illustrate this, Fig. 3C plots σ versus μ/*k*_B_*T* for four different temperatures. The solid curves, generated by using only these four global parameters, show excellent agreement with the data.

We now address the effect of a bandgap. We hypothesize that gap-induced changes in transport scattering times are dictated by changes to the carrier density and group velocity rather than changes to the universal electron-hole coupling strength α_0_. In this case, both terms in Eq. 1 are suitably modified. The thermally activated carrier densities *n_e_* and *n_h_* become functions of both μ/*k*_B_*T* and Δ/*k*_B_*T*, and we find that τ(Δ) is obtained from the gapless τ by a multiplicative function of Δ/*k*_B_*T* (see section 5.3). Because for μ = 0, *k*_B_*T* and Δ are the only remaining energy scales [the Coulomb energy drops out because it is present in both σ*_eh_*(Δ) and σ_0_], the normalized conductivity for the model hyperbolic band structure collapses as a function of Δ/*k*_B_*T*

$$\begin{array}{c} \frac{\sigma_{\mathrm{eh}}(\Delta )}{\sigma_{0}}=1+\frac{1}{\text{log}\;(2)}[\text{log}\;(\text{cosh}\;(\frac{\Delta }{4k_{B}T}))-\\ \frac{\Delta }{4k_{B}T}\text{tanh}\;(\frac{\Delta }{4k_{B}T})-\frac{1}{8}{\left(\frac{\Delta }{k_{B}T}\right)}^{2}\text{exp}\;(-\frac{5\Delta }{8k_{B}T})] \end{array}$$ (2)

**Fig. 4.**
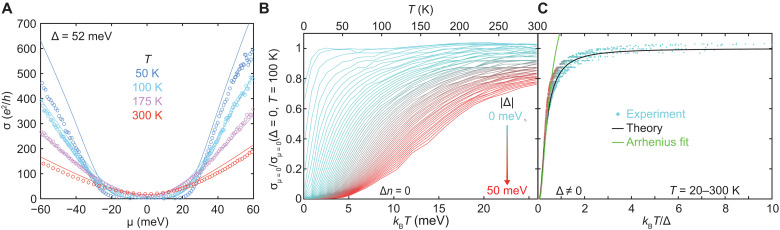
Hydrodynamic semiconductor. (**A**) Representative gapped conductivity measurements (symbols) as a function of μ (meV) for *T* = 50,100,175, and 300 K. The data are in excellent agreement with hydrodynamic theory developed in this work (solid lines). The same set of four global fit parameters has been used consistently to fit the full range of carrier densities, temperature, and bandgaps (additional data for Δ = 13,28, and 36 meV are shown in the Supplementary Materials). (**B**) Normalized charge-neutral conductivity as a function of *k*_B_*T* (lower *x* axis) and *T* (upper *x* axis) for varying Δ. The color gradient denotes the magnitude of ∣Δ∣. (**C**) Normalized charge-neutral conductivity as a function of *k*_B_*T*/Δ for temperatures from 20 to 300 K. The data collapse onto a single curve are in agreement with the theoretical prediction (solid line) of Eq. 2. The color scale for the data in (C) matches that in (B).

This temperature-mediated insulating-to-conducting crossover function is completely different from the usual Arrhenius behavior σ ∼ exp (−Δ/2*k*_B_*T*) seen in conventional disorder-limited semiconductors within the gap (although it mimics Arrhenius behavior at the lowest temperature). While this crossover function is specific to our model of two hyperbolic bands, it is only slightly modified for different band structures (see section S6.2 for details). Making use of the relationship between the top and bottom gates and Δ (see Materials and Methods), we plot the resulting function of *k*_B_*T*/Δ (solid line) in Fig. 4C alongside the experimental data (dots) of Fig. 4B (omitting the *T* < 20 K portion that lies in the impurity-limited regime) replotted as a function of *k*_B_*T*/Δ. As predicted, the experimental data collapse onto a single curve. The collapse of the experimental data validates our assumptions about α_0_ and provides strong evidence that transport in bilayer graphene remains electron-hole–limited even as we move deep into the insulating regime.

**Fig. 5.**
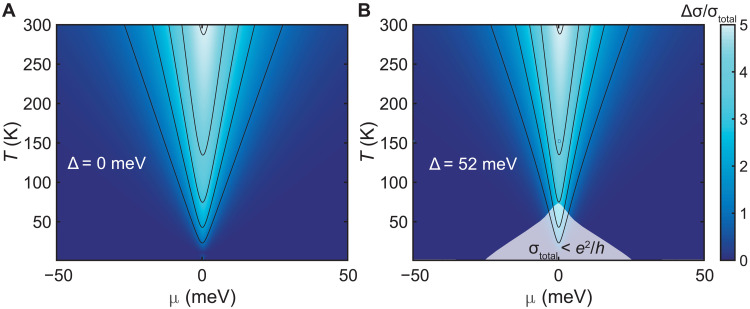
Phase space for hydrodynamic conductivity. Calculated ratio of Δσ = σ_ac + *i*_ − σ_total_ to σ_total_, for Δ = 0 (**A**) and 52 meV (**B**). Contour lines demarcate integer values, incrementing from 1. The shaded area in (B) shows the insulating regime where σ_total_ < *e*^2^/*h*. The degree to which electron-hole scattering dominates transport remains unchanged by the bandgap.

Having validated the dissipative hydrodynamics model, it is now possible to quantitatively map out the phase space for hydrodynamic conductivity. To do this, we calculate the net effect of electron-hole scattering by subtracting the conductivity (Eq. 1) from the conductivity calculated with only phonon and impurity scattering. We plot the ratio of this value to the total conductivity in Fig. 5, for the zero-gap case and for the case with Δ = 52 meV. As expected, electron-hole interactions dominate transport near charge neutrality—even in the presence of a bandgap—with the regions of dominance expanding as temperature increases.

Our results confirm that an intuitive model (as captured in Eq. 1) provides a complete description of the conductivity of bilayer graphene over a wide range of temperatures, carrier densities, and gap sizes. Our ultraclean samples are dominated by electron-hole scattering achieving both the first room-temperature hydrodynamic conductor including confirmation of Planckian dissipation and the first realization of a hydrodynamic semiconductor whose properties do not depend on material-specific parameters like the effective mass. We emphasize that our hydrodynamic theory can be easily adapted to other systems with different band structures, electron-phonon coupling, or disorder. For example, we find that the hydrodynamic conductivity seen here is suppressed for *n*_imp_ = 10^11^ cm^−2^ and disappears completely for *n*_imp_ = 10^12^ cm^−2^. The need for low disorder explains why the hydrodynamic regime went unexplored for so long: The required disorder level of *n*_imp_ ∼ 10^10^ cm^−2^ (i.e., τ_dis_ ∼ 4 ps) is only achievable in suspended samples (dielectric constant κ ∼ 1) or hBN-encapsulated samples with graphite gates (κ ∼ 4). However, once strong hydrodynamics is achieved (i.e., τ_dis_ ≫ τ_0_ and m*(*e*^2^/κ)^2^/*T* ≫ 1), then the hydrodynamic properties will be universal and material independent (see the Supplementary Materials). We therefore expect that these insights should be applicable to many ambipolar two-dimensional systems with low disorder and strong electron-hole interactions including gapped monolayer graphene, twisted bilayer graphene, narrow-gap semiconductors and semimetals, and optically excited electron-hole fluids.

As a room-temperature hydrodynamic conductor, bilayer graphene is an ideal model system for studying more complex hydrodynamic behavior—including effects of viscosity, flow through constrictions, collective sound modes, high-frequency magnetotransport, and shockwaves in supersonic flow—via a variety of techniques. Specifically, our experimentally measured values for the electron-hole scattering allow us to conclude that this platform should host more than a factor of 2 larger violation of the Wiedemann-Franz law compared to monolayer graphene (*19*), a large frequency window where one might observe electron-hole sound waves at temperatures extending to room temperature and beyond (*28*), and an ideal system to explore the recently found hydrodynamic spin generation effect (*29*) for applications in semiconductor spintronics, thereby combining semiconductor physics with viscous electronics.

## MATERIALS AND METHODS

Heterostructure devices were fabricated with the van der Waals assembly technique (*30*). To briefly summarize, a transfer substrate of polypropylene carbonate–coated polydimethylsiloxane is used to pick up the top layer of exfoliated hBN, which is then used to pick up the subsequent layers of the heterostructure. Once the heterostructure is assembled, it is transferred to the substrate of interest and annealed in vacuum (∼10^−8^ Torr) at 350°C. Depending on the gating and contact configurations of interest, different processing steps of electron beam lithography (NanoBeam nB4), etch, and electron beam evaporation are used to etch and define the heterostructure into a dual gated device with multiple terminals for Hall measurements, as outlined in the Supplementary Materials. Devices were then wire-bonded to a dual-inline package for measurement. An optical image of one device is shown in fig. S1.

Low-temperature to room-temperature measurements were measured in liquid helium cryogenic systems capable of temperatures as low as 1.2 K and magnetic fields as high as 14 T. High-temperature measurements were done in a cryostat with a heating stage for elevated temperatures. Device gates were biased with Keithley 2400 and Yokogawa GS200 DC source meters. The device current and voltages were measured with Stanford Research System 830 lock-in amplifiers. The conductivity measurements are performed at currents ∼10 to 100 nA, well within the range in which electrons may be considered to be in thermal equilibrium with the lattice even in the presence of strong electron-hole scattering (*19*).

For the dual-gated devices used in this study, Δ and μ can be independently controlled if the top and bottom gate capacitances are known. Therefore, we characterize the device by mapping the resistance as a function of top and bottom gate voltages (fig. S2A). The peak at μ = 0 follows a diagonal line whose slope is the ratio of the two capacitances. This is combined with Hall effect measurements to determine each capacitance individually, allowing us to define two experimental parameters: the interlayer potential energy difference Δ_ext_, which sets Δ; and an effective voltage *V*_eff_, which tunes μ at constant Δ_ext_. For the range considered in this work, Δ_ext_ ≈ 2.6Δ as determined experimentally from Arrhenius fittings, in good agreement with tight-binding models (*31*). The inset in fig. S2D shows the induced carrier density Δ*n* determined from low-temperature Hall effect measurements, taken along contours of fixed Δ_ext_ = 0,150 meV, as depicted in fig. S2C. These measurements confirm that (i) Δ*n* increases linearly with *V*_eff_; (ii) the samples are in the low-disorder limit with charge disorder below ∼3 × 10^10^cm^−2^; and (iii) a gap opens between the electron and hole branches for nonzero Δ_ext_. At higher temperatures, the Hall data show thermal excitation of electrons and holes (fig. S8). Details of device characterization and determination of carrier density, chemical potential, and bandgap are provided in the Supplementary Materials (sections S5.1 and S5.2).

## Supplementary Materials

### This PDF file includes:

Supplementary Text Figs. S1 to S15 Tables S1 and S2 References

## References

[1] R. N. Gurzhi, Hydrodynamic effects in solids at low temperature. Sov. Phys. Uspekhi 11, 255–270 (1968).

[2] B. N. Narozhny, I. V. Gornyi, M. Titov, M. Schütt, A. D. Mirlin, Hydrodynamics in graphene: Linear-response transport. Phys. Rev. B 91, 035414 (2015).

[3] M. Dyakonov, Shallow water analogy for a ballistic field effect transistor: New mechanism of plasma wave generation by dc current. Phys. Rev. Lett. 71, 2465–2468 (1993). 10054687 10.1103/PhysRevLett.71.2465

[4] H. Predel, H. Buhmann, L. W. Molenkamp, R. N. Gurzhi, A. N. Kalinenko, A. I. Kopeliovich, A. V. Yanovsky, Effects of electron-electron scattering on electron-beam propagation in a two-dimensional electron gas. Phys. Rev. B 62, 2057–2064 (2000).

[5] A. O. Govorov, J. J. Heremans, Hydrodynamic effects in interacting fermi electron jets. Phys. Rev. Lett. 92, 026803 (2004). 14753953 10.1103/PhysRevLett.92.026803

[6] M. Müller, L. Fritz, S. Sachdev, Quantum-critical relativistic magnetotransport in graphene. Phys. Rev. B 78, 115406 (2008).

[7] M. Müller, J. Schmalian, L. Fritz, Graphene: A nearly perfect fluid. Phys. Rev. Lett. 103, 025301 (2009). 19659217 10.1103/PhysRevLett.103.025301

[8] C. B. Mendl, M. Polini, A. Lucas, Coherent terahertz radiation from a nonlinear oscillator of viscous electrons. Appl. Phys. Lett. 118, 013105 (2021).

[9] J. Zaanen, Electrons go with the flow in exotic material systems. Science 351, 1026–1027 (2016). 26941303 10.1126/science.aaf2487

[10] D. Y. H. Ho, I. Yudhistira, N. Chakraborty, S. Adam, Theoretical determination of hydrodynamic window in monolayer and bilayer graphene from scattering rates. Phys. Rev. B 97, 121404 (2018).

[11] M. Zarenia, T. B. Smith, A. Principi, G. Vignale, Breakdown of the Wiedemann-Franz law in *AB*-stacked bilayer graphene. Phys. Rev. B 99, 161407 (2019).

[12] G. Wagner, D. X. Nguyen, S. H. Simon, Transport in bilayer graphene near charge neutrality: Which scattering mechanisms are important? Phys. Rev. Lett. 124, 026601 (2020). 32004029 10.1103/PhysRevLett.124.026601

[13] P. Gallagher, C.-S. Yang, T. Lyu, F. Tian, R. Kou, H. Zhang, K. Watanabe, T. Taniguchi, F. Wang, Quantum-critical conductivity of the Dirac fluid in graphene. Science 364, 158–162 (2019). 30819930 10.1126/science.aat8687

[14] Y. Nam, D.-K. Ki, D. Soler-Delgado, A. F. Morpurgo, Electron-hole collision limited transport in charge-neutral bilayer graphene. Nat. Phys. 13, 1207–1214 (2017).

[15] D. X. Nguyen, G. Wagner, S. H. Simon, Quantum boltzmann equation for bilayer graphene. Phys. Rev. B 101, 035117 (2020).

[16] C. A. Kukkonen, P. F. Maldague, Electron-hole scattering and the electrical resistivity of the semimetal TiS_2_. Phys. Rev. Lett. 37, 782–785 (1976).

[17] P. A. Lee, A. D. Stone, Universal conductance fluctuations in metals. Phys. Rev. Lett. 55, 1622–1625 (1985). 10031872 10.1103/PhysRevLett.55.1622

[18] D. A. Bandurin, I. Torre, R. K. Kumar, M. B. Shalom, A. Tomadin, A. Principi, G. H. Auton, E. Khestanova, K. S. Novoselov, I. V. Grigorieva, L. A. Ponomarenko, A. K. Geim, M. Polini, Negative local resistance caused by viscous electron backflow in graphene. Science 351, 1055–1058 (2016). 26912363 10.1126/science.aad0201

[19] J. Crossno, J. K. Shi, K. Wang, X. Liu, A. Harzheim, A. Lucas, S. Sachdev, P. Kim, T. Taniguchi, K. Watanabe, T. A. Ohki, K. C. Fong, Observation of the Dirac fluid and the breakdown of the Wiedemann-Franz law in graphene. Science 351, 1058–1061 (2016). 26912362 10.1126/science.aad0343

[20] A. I. Berdyugin, S. G. Xu, F. M. D. Pellegrino, R. K. Kumar, A. Principi, I. Torre, M. B. Shalom, T. Taniguchi, K. Watanabe, I. V. Grigorieva, M. Polini, A. K. Geim, D. A. Bandurin, Measuring Hall viscosity of graphene’s electron fluid. Science 364, 162–165 (2019). 30819929 10.1126/science.aau0685

[21] C. R. Dean, A. F. Young, I. Meric, C. Lee, L. Wang, S. Sorgenfrei, K. Watanabe, T. Taniguchi, P. Kim, K. L. Shepard, J. Hone, Boron nitride substrates for high-quality graphene electronics. Nat. Nanotechnol. 5, 722–726 (2010). 20729834 10.1038/nnano.2010.172

[22] J. K. Viljas, T. T. Heikkilä, Electron-phonon heat transfer in monolayer and bilayer graphene. Phys. Rev. B 81, 245404 (2010).

[23] X. Li, K. M. Borysenko, M. B. Nardelli, K. W. Kim, Electron transport properties of bilayer graphene. Phys. Rev. B 84, 195453 (2011).

[24] M. Lv, S. Wan, Screening-induced transport at finite temperature in bilayer graphene. Phys. Rev. B 81, 195409 (2010).

[25] M. Kohler, Allgemeine theorie der abweichungen von der mathiessenschen regel. Z. Phys. 126, 495–506 (1949).

[26] J. Zaanen, Why the temperature is high. Nature 430, 512–513 (2004). 15282588 10.1038/430512a

[27] K. Zou, X. Hong, J. Zhu, Effective mass of electrons and holes in bilayer graphene: Electron-hole asymmetry and electron-electron interaction. Phys. Rev. B 84, 085408 (2011).

[28] T. V. Phan, J. C. W. Song, L. S. Levitov, Ballistic Heat Transfer and Energy Waves in an Electron System. arXiv:1306.4972 (2013).

[29] R. Takahashi, M. Matsuo, M. Ono, K. Harii, H. Chudo, S. Okayasu, J. Ieda, S. Takahashi, S. Maekawa, E. Saitoh, Spin hydrodynamic generation. Nat. Phys. 12, 52–56 (2016).

[30] L. Wang, I. Meric, P. Y. Huang, Q. Gao, Y. Gao, H. Tran, T. Taniguchi, K. Watanabe, L. M. Campos, D. A. Muller, J. Guo, P. Kim, J. Hone, K. L. Shepard, C. R. Dean, One-dimensional electrical contact to a two-dimensional material. Science 342, 614–617 (2013). 24179223 10.1126/science.1244358

[31] E. McCann, Asymmetry gap in the electronic band structure of bilayer graphene. Phys. Rev. B 74, 161403 (2006).

[32] K. M. Borysenko, J. T. Mullen, X. Li, Y. G. Semenov, J. M. Zavada, M. B. Nardelli, K. W. Kim, Electron-phonon interactions in bilayer graphene. Phys. Rev. B 83, 161402 (2011).

[33] J. Huang, J. A. Alexander-Webber, T. J. B. M. Janssen, A. Tzalenchuk, T. Yager, S. Lara-Avila, S. Kubatkin, R. L. Myers-Ward, V. D. Wheeler, D. K. Gaskill, R. J. Nicholas, Hot carrier relaxation of dirac fermions in bilayer epitaxial graphene. J. Phys. Condens. Matter 27, 164202 (2015). 25835029 10.1088/0953-8984/27/16/164202

[34] D. K. Efetov, P. Kim, Controlling electron-phonon interactions in graphene at ultrahigh carrier densities. Phys. Rev. Lett. 105, 256805 (2010). 21231611 10.1103/PhysRevLett.105.256805

[35] S. Das Sarma, S. Adam, E. H. Hwang, E. Rossi, Electronic transport in two-dimensional graphene. Rev. Mod. Phys. 83, 407–470 (2011).

[36] P. S. Alekseev, A. P. Dmitriev, I. V. Gornyi, V. Y. Kachorovskii, B. N. Narozhny, M. Schütt, M. Titov, Magnetoresistance of compensated semimetals in confined geometries. Phys. Rev. B 95, 165410 (2017).

[37] E. McCann, M. Koshino, The electronic properties of bilayer graphene. Rep. Prog. Phys. 76, 056503 (2013). 23604050 10.1088/0034-4885/76/5/056503

[38] E. McCann, V. I. Fal’ko, Landau-level degeneracy and quantum Hall effect in a graphite bilayer. Phys. Rev. Lett. 96, 086805 (2006). 16606214 10.1103/PhysRevLett.96.086805

[39] H. Ochoa, E. V. Castro, M. I. Katsnelson, F. Guinea, Temperature-dependent resistivity in bilayer graphene due to flexural phonons. Phys. Rev. B 83, 235416 (2011).

[40] D. Svintsov, V. Vyurkov, S. Yurchenko, T. Otsuji, V. Ryzhii, Hydrodynamic model for electron-hole plasma in graphene. J. Appl. Phys. 111, 083715 (2012).

[41] K. Kaasbjerg, K. S. Thygesen, K. W. Jacobsen, Unraveling the acoustic electron-phonon interaction in graphene. Phys. Rev. B 85, 165440 (2012).

[42] T. Sohier, M. Calandra, C.-H. Park, N. Bonini, N. Marzari, F. Mauri, Phonon-limited resistivity of graphene by first-principles calculations: Electron-phonon interactions, strain-induced gauge field, and Boltzmann equation. Phys. Rev. B 90, 125414 (2014).

[43] M. Combescot, R. Combescot, Conductivity relaxation time due to electron-hole collisions in optically excited semiconductors. Phys. Rev. B 35, 7986–7992 (1987). 10.1103/physrevb.35.7986 9941133

[44] J. Schiefele, F. Sols, F. Guinea, Temperature dependence of the conductivity of graphene on boron nitride. Phys. Rev. B 85, 195420 (2012).

[45] S. Fratini, F. Guinea, Substrate-limited electron dynamics in graphene. Phys. Rev. B 77, 195415 (2008).

[46] V. Perebeinos, P. Avouris, Inelastic scattering and current saturation in graphene. Phys. Rev. B 81, 195442 (2010).

[47] M. Polini, G. Vignale, V. Pellegrini, J. K. Jain, The quasiparticle lifetime in a doped graphene sheet, in *No-Nonsense Physicist: An Overview of Gabriele Giuliani’s Work and Life* (Scuola Normale Superiore, 2016), pp. 107–124.

[48] S. Adam, E. H. Hwang, V. M. Galitski, S. D. Sarma, A self-consistent theory for graphene transport. Proc. Natl. Acad. Sci. U.S.A. 104, 18392–18397 (2007). 18003926 10.1073/pnas.0704772104 PMC2141788

[49] D. Rhodes, S. H. Chae, R. Ribeiro-Palau, J. Hone, Disorder in van der waals heterostructures of 2d materials. Nat. Mater. 18, 541–549 (2019). 31114069 10.1038/s41563-019-0366-8

[50] M. Müller, S. Sachdev, Collective cyclotron motion of the relativistic plasma in graphene. Phys. Rev. B 78, 115419 (2008).

[51] X.-F. Wang, T. Chakraborty, Coulomb screening and collective excitations in biased bilayer graphene. Phys. Rev. B 81, 081402 (2010).

[52] L. Jiang, Z. Shi, B. Zeng, S. Wang, J.-H. Kang, T. Joshi, C. Jin, L. Ju, J. Kim, T. Lyu, Y.-R. Shen, M. Crommie, H.-J. Gao, F. Wang, Soliton-dependent plasmon reflection at bilayer graphene domain walls. Nat. Mater. 15, 840–844 (2016). 27240109 10.1038/nmat4653

[53] N. Ashcroft, N. Mermin, *Solid State Physics* (Saunders College, 1976).

[54] K. von Klitzing, The quantized hall effect. Rev. Mod. Phys. 58, 519–531 (1986).

[55] B. L. Altshuler, A. G. Aronov, D. E. Khmelnitsky, Effects of electron-electron collisions with small energy transfers on quantum localisation. J. Phys. C 15, 7367–7386 (1982).

[56] A. K. Geim, K. S. Novoselov, The rise of graphene. Nat. Mater. 6, 183–191 (2007). 17330084 10.1038/nmat1849

[57] J. A. N. Bruin, H. Sakai, R. S. Perry, A. P. Mackenzie, Similarity of scattering rates in metals showing T-linear resistivity. Science 339, 804–807 (2013). 23413351 10.1126/science.1227612

[58] H.-Y. Xie, M. S. Foster, Transport coefficients of graphene: Interplay of impurity scattering, Coulomb interaction, and optical phonons. Phys. Rev. B 93, 195103 (2016).

