# Supplementary material for: Dissipation-enabled hydrodynamic conductivity in a tunable bandgap semiconductor
Source: Sci Adv. 2022 Apr 15;8(15):eabi8481. doi: 10.1126/sciadv.abi8481 (PMC9012458; doi:10.1126/sciadv.abi8481)
Supplement: Supplementary file 1 — Supplementary Text Figs. S1 to S15 Tables S1 and S2 References [file sciadv.abi8481_sm.pdf]

Science Advances
AAAS

Supplementary Materials for

Dissipation-enabled hydrodynamic conductivity in a tunable bandgap semiconductor

Cheng Tan, Derek Y. H. Ho, Lei Wang, Jia I. A. Li, Indra Yudhistira, Daniel A. Rhodes, Takashi Taniguchi, Kenji Watanabe, Kenneth Shepard, Paul L. McEuen, Cory R. Dean, Shaffique Adam*, James Hone*

*Corresponding author. Email: shaffique.adam@yale-nus.edu.sg (S.A.); jh2228@columbia.edu (J.H.)

Published 15 April 2022, *Sci. Adv.* **8**, eabi8481 (2022)
DOI: 10.1126/sciadv.abi8481

This PDF file includes:

Supplementary Text
Figs. S1 to S15
Tables S1 and S2
References

1 Fabrication of Devices

Of the five total devices measured in this work, four were made with graphite gates, and one with metallic gates. All showed closely similar behavior. To fabricate dual graphite gate devices, we first assemble a stack with hBN, top graphite, hBN, graphite contacts (optional), BLG, hBN, and bottom graphite in that order. The stack is then etched twice with 40 sccm CHF_3 + 4 sccm O_2 to first shape the top gate and then the channel; Cr/Pd/Au (2 nm/40 nm/50 nm) is evaporated to make contact to the gates and channel. The metallic gate device was made by placing down a hBN, BLG, hBN stack on to a pre-patterned Pd back gate. Cr/Pd/Au (2 nm/20 nm/50 nm) was evaporated as top gate before the stack was then etched to shape the device. Finally, Cr/Pd/Au (2 nm/20 nm/50 nm) was evaporated to make contacts to the channel. We list in Table 1 the device dimensions and figures they correspond to. A typical device with dual graphite gates and graphite contacts is shown in Fig. S1, and the cross section schematic is presented in Fig. S2B.

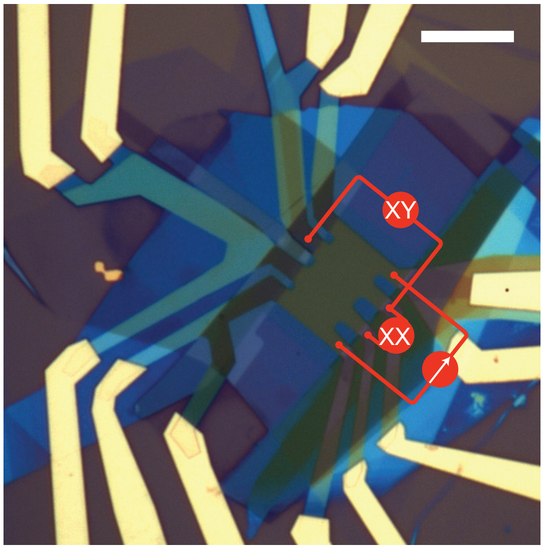

Figure S1: **Device schematic.** A fabricated device with dual graphite gates and graphite contacts. The measurement scheme is shown in red. Scale bar is 10 μm.

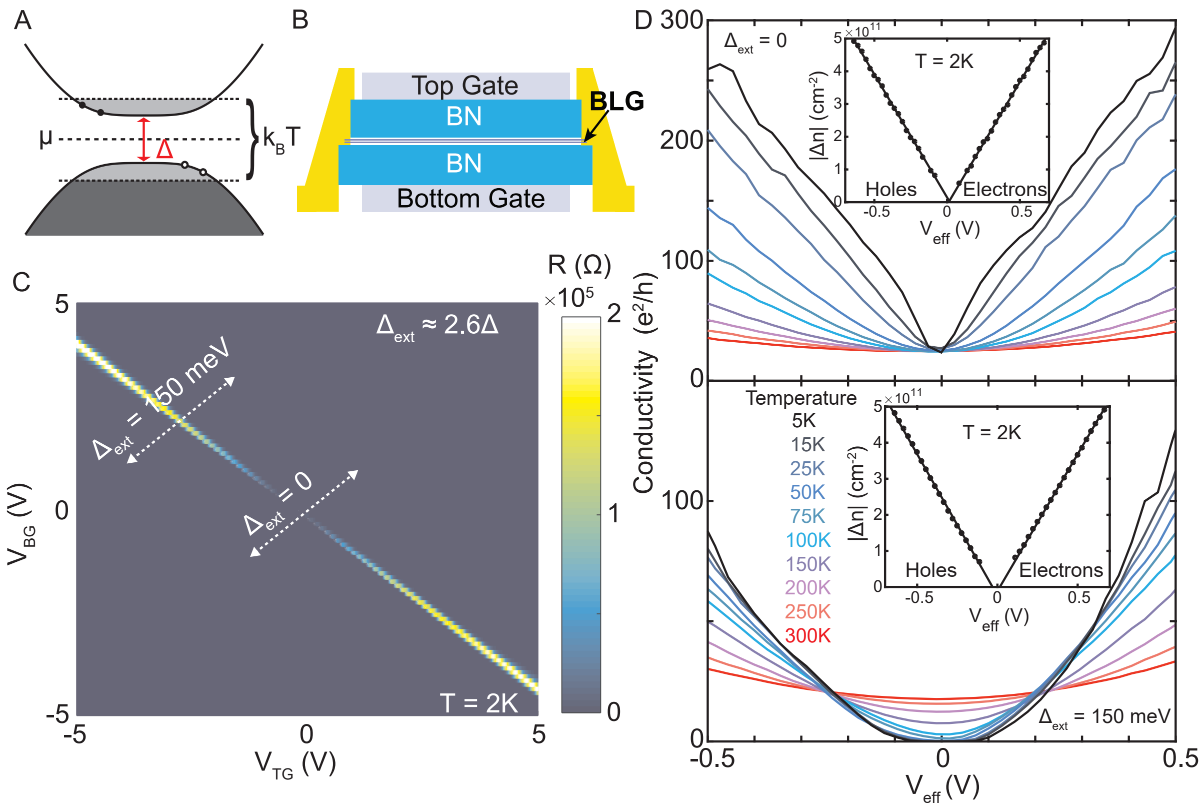

Figure S2: **Experimental characterization of dual-gated bilayer graphene.** (A) Schematic bandstructure consisting of two hyperbolic bands separated by a tunable band gap Δ, with chemical potential μ and thermal energy $k_B T$. (B) Cross sectional schematic of an encapsulated BLG device with dual local gates. (C) Resistance as a function of independent top and bottom gate voltages V_{TG} and V_{BG}. Dashed lines show cuts at Δ_{ext} = 0 and 150 meV. (D) Measured conductivity as a function of V_{eff} at $\Delta_{\mathrm{ext}} = 0$ (top) and $\Delta_{\mathrm{ext}} = 150$ meV (bottom) for T from 5K to 300K. Insets show the Hall density.

Table 1: **Device Parameters.** List of measured devices and their corresponding figures in the main text.

Device	Width (um)	Length (um)	Gates	Contacts	Figures
1	2	11	Graphite	Metallic	2A
2	5.5	3.5	Graphite	Graphite	2A
3	6.26	10.5	Graphite	Metallic	2A-B, 3, 4
4	8	8	Graphite	Metallic	NA
5	9.6	3.85	Metallic	Metallic	NA

2 Two-fluid model for bilayer graphene in the hydrodynamic regime

The transport properties of bilayer graphene in the hydrodynamic can be determined using a two-fluid model (see e.g. Ref. (*12, 15*)). The two-fluid model describes the evolution of the average drift velocity of electrons and holes $\vec{u}_{e/h}$. It consists of two equations of motion, one for each carrier species:

$$\begin{aligned} \frac{d\vec{u}_e}{dt} &= -\frac{\vec{u}_e - \vec{u}_h}{\tau_{\text{e,eh}}} - \frac{\vec{u}_e}{\tau_{\text{e,dis}}} - \frac{e\vec{E}}{m_e^*}, \\ \frac{d\vec{u}_h}{dt} &= -\frac{\vec{u}_h - \vec{u}_e}{\tau_{\text{h,eh}}} - \frac{\vec{u}_h}{\tau_{\text{h,dis}}} + \frac{e\vec{E}}{m_h^*}, \end{aligned} \tag{S1}$$

where $e > 0$ is the magnitude of the electron charge. Here, $\tau^{-1}_{e(h),eh}$ is the average rate of collisions with holes (electrons) per electron (hole), while $\tau^{-1}_{e(h),\text{dis}}$ is the average rate of collisions with surrounding impurities and phonons per electron (hole). $m^*_{e/h}$ are the effective masses and $\vec{E}$ is an external electric field. We detail the method for calculating the various relaxation times $\tau_{e/h}$ in the subsection below. The electron and hole drift velocities $\vec{u}_{e,h}$ are the average velocities of the electrons and holes and given by

$$\vec{u}_{e/h} = \left(\int \frac{d^2k}{(2\pi)^2} \frac{\hbar\vec{k}}{m^*_{e/h}} f_{e/h}(\epsilon_{\vec{k}}) \right) \Big/ \left(\int \frac{d^2k}{(2\pi)^2} f_{e/h}(\epsilon_{\vec{k}}) \right), \tag{S2}$$

where $f(\epsilon)$ is the standard Fermi distribution function $1/\left(\exp[(\epsilon - \mu)/(k_BT)] + 1\right)$, with $\epsilon_{\vec{k}} = \hbar^2 k^2/2m^*$, and the integrals are over the entire Brillouin zone. The above equations are obtained from the Boltzmann kinetic equations using standard techniques (*12*). Physically, they describe the electrons (holes) as moving with one effective drift velocity $\vec{u}_{e(h)}$ This description works well in the hydrodynamic regime where the electron-electron collision rate is the largest scattering rate in the system. It is likely to hold even when electron-hole scattering rate is larger than but still comparable to that of electron-electron (*36*).

Note that since electron-hole collisions preserve total momentum density, $m_e^* n_e d\vec{u}_e/dt + m_h^* n_h d\vec{u}_h/dt = 0$ must be true in the absence of external forces (i.e. $\tau_{e/h,\text{dis}}^{-1} = 0$ and $\vec{E} = 0$). Combining equations (S1) with this condition yields this constraint on electron-hole relaxation times: $n_e m_e^* \tau_{h,eh} = n_h m_h^* \tau_{e,eh}$. Taking $\tau_{e,eh} = \frac{m_e^* n_e + m_h^* n_h}{m_h^* n_h} \tau_0$ and $\tau_{h,eh} = \frac{m_e^* n_e + m_h^* n_h}{m_e^* n_e} \tau_0$ ensures that the constraint is satisfied, where $\tau_0^{-1} \equiv \tau_{e,eh}^{-1} + \tau_{h,eh}^{-1}$ evaluated at charge neutrality $n_e = n_h$. To obtain the conductivity, one may solve Eqs. (S1) for the steady-state $\vec{u}_{e/h}$, substitute these into the total current density $\vec{j} = n_e(-e)\vec{u}_e + n_h e \vec{u}_h$, and read off the conductivity σ in $\vec{j} = \sigma \vec{E}$. It is more instructive however to work instead in terms of the center-of-mass (COM) velocity

$$\vec{\bar{u}} \equiv \frac{n_e m_e^* \vec{u}_e + n_h m_h^* \vec{u}_h}{n_e m_e^* + n_h m_h^*} \tag{S3}$$

and the relative velocity

$$\vec{v} = \vec{u}_e - \vec{u}_h. \tag{S4}$$

Using these variables, current density becomes

$$\vec{j} = -\frac{n_e n_h}{n_e m_e^* + n_h m_h^*}(m_e^* + m_h^*) e \vec{v} + (n_h - n_e) e \vec{\bar{u}}, \tag{S5}$$

in which the first term represents the contribution from the electrons and holes moving in opposite direction due to the opposite forces exerted on them by the electric field and the second represents the contribution from electrons and holes moving in unison in the same direction due to the Coulomb drag "friction" between electrons and holes, the strength of which is quantified by τ_0^{-1}. Rewriting Eqs. (S1) in terms of $\vec{\bar{u}}$ and $\vec{v}$ and performing rearrangements to make the

time-derivatives of $\vec{u}$ and $\vec{v}$ the subjects, we find

$$\frac{d\vec{u}}{dt} = \left[-\vec{u}\left(\frac{n_e m_e^*}{\tau_{e,\text{dis}}} + \frac{n_h m_h^*}{\tau_{h,\text{dis}}}\right) - \frac{n_e m_e^* n_h m_h^*}{n_e m_e^* + n_h m_h^*}\left(\frac{1}{\tau_{e,\text{dis}}} - \frac{1}{\tau_{h,\text{dis}}}\right)\vec{v} - e\vec{E}\left(n_e - n_h\right)\right]\left(n_e m_e^* + n_h m_h^*\right)^{-1}, \tag{S6}$$

$$\frac{d\vec{v}}{dt} = -\frac{\vec{v}}{\tau_0} - \vec{u}\left(\frac{1}{\tau_{e,\text{dis}}} - \frac{1}{\tau_{h,\text{dis}}}\right) - \frac{\vec{v}}{n_e m_e^* + n_h m_h^*}\left(\frac{n_h m_h^*}{\tau_{e,\text{dis}}} + \frac{n_e m_e^*}{\tau_{h,\text{dis}}}\right) - e\vec{E}\left(\frac{1}{m_e^*} + \frac{1}{m_h^*}\right), \tag{S7}$$

where we have made use of relationships above. Several insights may be drawn here. From Eq. (S6), the COM velocity is unaffected by the electric field at charge neutrality $n_e = n_h$ since the net force from the electric field is zero. Away from neutrality, it increases without bound in the absence of external dissipative scattering mechanisms and the magnitude of $\vec{u}$ tends to infinity as $t \to \infty$. Put differently, there exists no steady-state (i.e. time-independent) solution $\vec{u}$ satisfying Eq. (S6) with $\tau_{e,\text{dis}}^{-1} = \tau_{h,\text{dis}}^{-1} = 0$ and $d\vec{u}/dt = 0$. From Eq. (S7) on the other hand, the relative velocity is finite even when $\tau_{e,\text{dis}}^{-1} = \tau_{h,\text{dis}}^{-1} = 0$, since it still admits a steady-state solution $\vec{v} = -e\vec{E}\tau_0(1/m_e^* + 1/m_e^*)$. Noting that

$$\vec{u}_{e/h} = \vec{u} \pm \frac{n_{h/e} m_{h/e}^*}{n_e m_e^* + n_h m_h^*}\vec{v}, \tag{S8}$$

the above statements together imply that current density $\vec{j}$ and conductivity are formally infinite in the absence of external momentum dissipation if $n_e \neq n_h$. It is only precisely at $n_e = n_h$ that the current density is finite even in the absence of external dissipation (i.e. electron-hole scattering alone can relax a net current) since the electric field is unable to accelerate the center of mass. Finally, we note from both Eqs. (S6) and (S7) that the COM and relative velocities are completely decoupled from each other when the dissipative relaxation times $\tau_{e,\text{dis}}$ and $\tau_{h,\text{dis}}$ are equal. In this case, changing the relative velocity $\vec{v}(t)$ whilst maintaining constant COM velocity $\vec{u}(t)$ leads to changes in the external dissipative frictional forces on electrons and holes respectively that exactly cancel each other.

Solving Eqs. (S6) and (S7) for the steady-state $\vec{u}$ and $\vec{v}$ by setting the time-derivatives to zero, we obtain

$$\vec{u} = -e\vec{E}\frac{(n_e\tau_{e,\mathrm{dis}} - n_h\tau_{h,\mathrm{dis}})\tau_0 + (n_e - n_h)\tau_{e,\mathrm{dis}}\tau_{h,\mathrm{dis}}}{(n_e m_e^* + n_h m_h^*)\tau_0 + n_e m_e^*\tau_{h,\mathrm{dis}} + n_h m_h^*\tau_{e,\mathrm{dis}}}, \tag{S9}$$

$$\vec{v} = -e\vec{E}\frac{(n_e m_e^* + n_h m_h^*)(m_e^*\tau_{h,\mathrm{dis}} + m_h^*\tau_{e,\mathrm{dis}})\tau_0}{m_e^* m_h^*\left[(n_e m_e^* + n_h m_h^*)\tau_0 + n_e m_e^*\tau_{h,\mathrm{dis}} + n_h m_h^*\tau_{e,\mathrm{dis}}\right]}. \tag{S10}$$

One may substitute these equations into Eq. (S5) and directly read off the conductivity from $\vec{j} = \sigma\vec{E}$. To simplify the expression, we make the common assumption of equal electron and hole effective masses $m_e^* = m_h^* \equiv m^*$, resulting in

$$\begin{aligned}\vec{j} = & \left[\frac{e^2}{m^*}\frac{2n_e n_h\tau_0(\tau_{e,\mathrm{dis}} + \tau_{h,\mathrm{dis}})}{n_e(\tau_0 + \tau_{h,\mathrm{dis}}) + n_h(\tau_0 + \tau_{e,\mathrm{dis}})}\right]\vec{E} \\ & + \left[\frac{e^2}{m^*}\frac{(n_h - n_e)\left(n_h\tau_{h,\mathrm{dis}}(\tau_0 + \tau_{e,\mathrm{dis}}) - n_e\tau_{e,\mathrm{dis}}(\tau_0 + \tau_{h,\mathrm{dis}})\right)}{n_h(\tau_0 + \tau_{e,\mathrm{dis}}) + n_e(\tau_0 + \tau_{h,\mathrm{dis}})}\right]\vec{E},\end{aligned} \tag{S11}$$

where the first and second terms represent contributions from the relative and COM motions respectively of the electron-hole plasma.

Thus far, our calculation has been exact and no approximations have been made. We now consider the limit of strong electron-hole scattering $\tau_0/\tau_{e,\mathrm{dis}}, \tau_0/\tau_{h,\mathrm{dis}} \to 0$, and Taylor expand the current density to zeroth order in $\tau_0/\tau_{e/h,\mathrm{dis}}$ and obtain $\vec{j} = \sigma_c\vec{E} + \sigma_{\mathrm{dis}}\vec{E}$, where

$$\sigma_c = \frac{e^2}{m^*}\frac{n_e n_h(n_e + n_h)(\tau_{e,dis}^{-1} + \tau_{h,dis}^{-1})^2}{\left(n_e\tau_{e,dis}^{-1} + n_h\tau_{h,dis}^{-1}\right)^2}\tau_0 \tag{S12}$$

represents the Coulomb drag conductivity arising from the Coulombic friction between electrons and holes, and

$$\sigma_{\mathrm{dis}} = \frac{e^2}{m^*}\frac{(n_e - n_h)^2}{n_e\tau_{e,dis}^{-1} + n_h\tau_{h,dis}^{-1}}. \tag{S13}$$

represents the conductivity due to external dissipative forces. Eqs. (S12) and (S13) are used for all the plots calculated in the main text.

Evidently from Eq. (S12), the drag conductivity σ_c is determined by electron-hole scattering time τ_0 and the *ratio* of electron and hole scattering times from external dissipative mechanisms

(i.e. if $\tau_{e,dis} = \tau_{h,dis}$, the dependence on both $\tau_{e,dis}$ and $\tau_{h,dis}$ vanishes). A particular exception is at the CNP $n_e = n_h$, at which the dissipative times drop out and σ_c depends only on τ_0. We find

$$\sigma_c = \sigma_0 \times \left(\frac{2}{\log(2)} \frac{\log(1+\exp(\mu/k_BT))\log(1+\exp(-\mu/k_BT))}{\log(1+\exp(\mu/k_BT))+\log(1+\exp(-\mu/k_BT))} \right), \tag{S14}$$

where $\sigma_0 = (e^2/h) \times 8\log(2)/\alpha_0$. Near charge neutrality, the term in large brackets above asymptotes to $\exp\left[-(1/3)\left(\mu/(k_BT)\right)^2\right]$, and this is what we show in the main text for simplicity since it is in good agreement with experimental data (See e.g. Fig. 3A).

3 Scattering times in gapless bilayer graphene

In this section we discuss the scattering times used in the main text. Electronic scattering times in bilayer graphene have been thoroughly studied for well over a decade and comprehensive reviews may be found in Refs. (*35, 37*). In this section we summarize the relevant aspects of the subject for our experiment.

Electrons in gapless bilayer graphene are well-described by a parabolic band dispersion at energies below 0.4 eV (*38*), corresponding to density $\sim 10^{13}$ cm^{-2} and temperature ~ 4600 K. The system has been studied experimentally in two configurations- mounted on a hexagonal boron nitride substrate or suspended between supports, the former of which is the focus of this work.

Experimental measurements of charge conductivity in hBN-supported samples have been explained in terms of charged impurity scattering (*21*) as well as in-plane acoustic phonons (*39*). More recently, samples of hBN-supported bilayer graphene were reported (*18*) to have such high levels of purity as to be in the hydrodynamic regime (*2, 10, 11*), in which the scattering rate of electrons with one another exceeds that with impurities and phonons. Here, we give a detailed comparison of the above mentioned scattering rates. We will in-turn discuss (i) Acoustic phonon scattering, (*22, 32, 39*) (ii) Optical phonons (*22, 23*) , (iii) Charged-impurity scattering (*35*), and

(iv) Electron-hole scattering (*12, 14, 40*) (note that the electron-electron scattering rate is not relevant (*12*) for current relaxation and is included only for completeness). Our comparison is summarized in Fig. S3, where we find that at low carrier density (n $\lesssim 3 \times 10^{11}\mathrm{cm}^{-2}$), for all temperatures considered (50-300K), electron-hole scattering dominates as the largest relevant (i.e. current-relaxing) scattering rate. The forms of the equations used to fit to the scattering rates from experimental data in the main text are consistent with those found in the large body of calculations in the literature on electron scattering rates in bilayer graphene (which we simply summarize and reproduce briefly below).

3.1 Acoustic phonon scattering

We calculate the scattering time for electron-acoustic-phonon scattering using the inverse quasi-particle lifetime (see e.g. Ref. (*22, 41*)). The quasiparticle lifetime $\tau(\epsilon_{k,\gamma})$ of a quasiparticle at energy $\epsilon_{k,\gamma}$ with acoustic phonons is given by

$$\frac{1}{\tau(\epsilon_{k,\gamma})} = \frac{2\pi}{\hbar}\int \frac{d^2q}{(2\pi)^2}|g_{\mathbf{k+q},\mathbf{k}}|^2 \times [(N_q + 1 - f(\epsilon_{k,\gamma} - \hbar v_s q))\,\delta(\epsilon_{\mathbf{k+q},\gamma} - \epsilon_{\mathbf{k},\gamma} + \hbar v_s q) + (f(\epsilon_{k,\gamma} + \hbar v_s q) + N_q)\,\delta(\epsilon_{\mathbf{k+q},\gamma} - \epsilon_{\mathbf{k},\gamma} - \hbar v_s q)], \quad \text{(S15)}$$

where

$$\epsilon_{k,\gamma} = \mathrm{sign}(\gamma)\frac{\hbar^2 k^2}{2m} \quad \text{(S16)}$$

with $\gamma = \pm 1$ for conduction and valence band respectively, $v_s = 13.6\mathrm{km\ s}^{-1}$ is the speed of sound for transverse acoustic phonons (*42*) and the deformation potential matrix element equals (*32*)

$$|g_{\mathbf{k+q},\mathbf{k}}|^2 = \frac{(D_{ac})^2\hbar q}{2\rho v_s}\left(\frac{1+\cos(2\theta_{\mathbf{k},\mathbf{k+q}})}{2}\right) \quad \text{(S17)}$$

with $D_{ac} =$ 10-30 eV according to estimates in the literature (*22, 39*) (we use $D_{ac} = 15$ eV for all our calculations in this section) and $\rho = 1.52 \times 10^{-24}\ \mathrm{kg/m^{-2}}$ is the areal mass density of

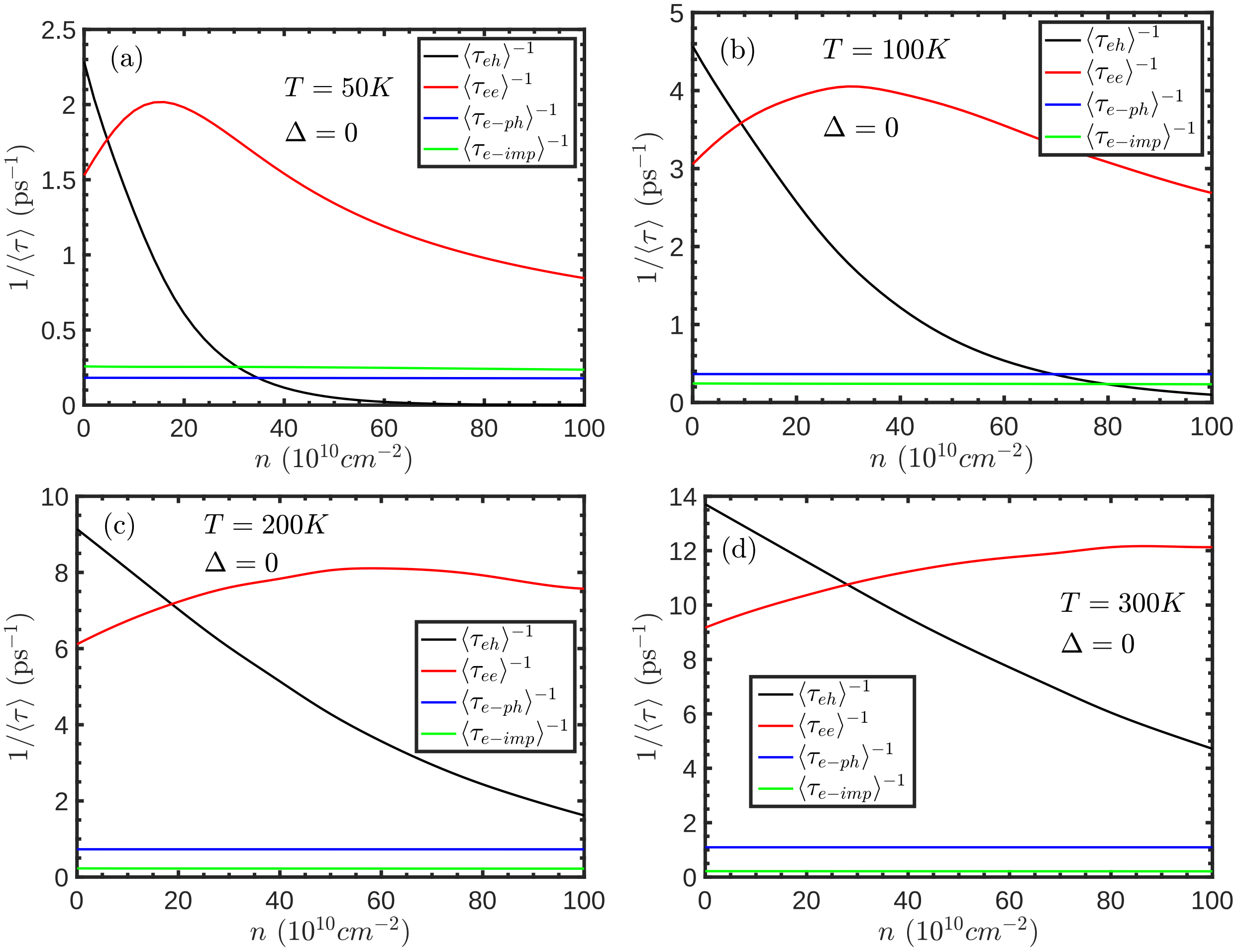

Figure S3: **Comparison of the relevant scattering rates**. Here we use $n_{\rm imp} = 10^{10}{\rm cm}^{-2}$, $D_{ac} = 15eV$ throughout (see text for details). A sizable hydrodynamic density window in which τ_{ee}^{-1} dominates occurs at all temperatures above 50K. At low densities, τ_{eh}^{-1} dominates over a window of densities that grows in size with temperature.

bilayer graphene. N_q is the Bose-Einstein distribution

$$N_q = \frac{1}{\exp(\frac{\hbar v_s q}{k_B T}) - 1} \tag{S18}$$

The scattering time $\tau\left(\epsilon_{k,\gamma}\right)$ above is easily computed numerically. To obtain a representative scattering rate for all the electrons relevant to charge transport (i.e. electrons within k_BT of the Fermi surface), we perform a thermal average (*43*) by inserting $\tau\left(\epsilon_{k,\gamma}\right)$ (with $\gamma = 1$ for

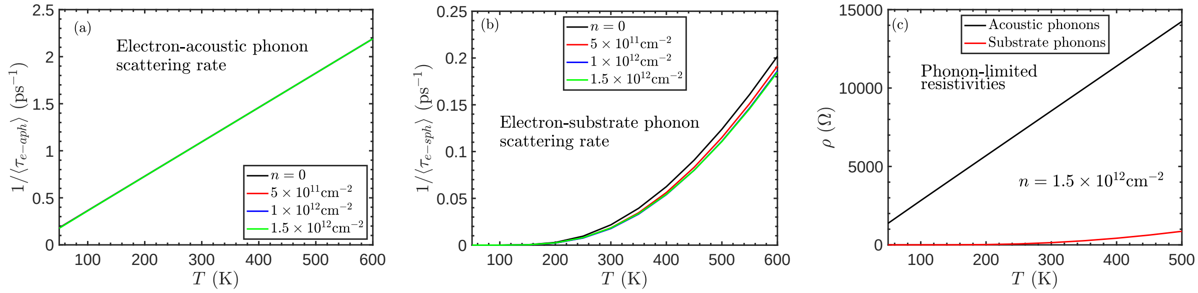

Figure S4: **Acoustic and optical phonon scattering**. Comparison of electron scattering rates with (a) acoustic phonons and (b) optical polar substrate phonons. The contributions to resistivity are estimated in (c) using the standard expression $\rho = m/(ne^2\langle\tau\rangle)$. Here we have used the same parameters for acoustic phonons as detailed in Sec. 3.1. For the substrate phonon calculations, we follow the parameters of Ref. (*44*). Namely, the zero, intermediate and high frequency dielectric constants are taken to be 5.09, 4.575 and 4.10, respectively, and the two polar optical phonon energies of hBN are taken to be 101.6 meV and 195.7 meV respectively.

electrons) into

$$\langle\tau\rangle = \frac{\int_0^\infty d\epsilon\tau(\pm\epsilon)|\epsilon|\left(-\frac{\partial f_{e/h}(\epsilon)}{\partial\epsilon}\right)}{\int_0^\infty d\epsilon|\epsilon|\left(-\frac{\partial f_{e/h}(\epsilon)}{\partial\epsilon}\right)}, \tag{S19}$$

where the $\pm\epsilon$ and e/h correspond to $\gamma = \pm 1$ for electrons and holes respectively, with $f_{e/h} = (\exp((\epsilon \pm \mu)/k_B T) + 1)^{-1}$.

We plot the inverse of the resulting thermally-averaged lifetime in Fig. S4(a) below as a function of temperature for different densities. Consistent with previous results in the literature (*22, 39*), we find that in the high temperature regime ($k_B T \geq \mu$), the scattering time (i.e. inverse lifetime) is $1/\tau\left(\epsilon_{k,\gamma}\right) = \alpha_{ac} k_B T/\hbar$, with the proportionality constant α_{ac} determined by the value taken for the deformation potential. The estimated range for the deformation potential based on the literature is $D_{ac} =$ 15-30 eV, corresponding to $\alpha_{ac} = 0.028$-0.11. In Fig. S3 we used $D_{ac} =$ 15 eV (or $\alpha_{ac} = 0.03$). In the main text, α_{ac} is left as a fitting parameter and we found 0.030 ± 0.008 for electrons and 0.041 ± 0.008 for holes (see Table 2 below) which are both well within the expected range.

3.2 Optical phonon scattering

The scattering rate of electrons with bilayer graphene optical phonons is three orders of magnitude lower than with acoustic phonons for all electron energies below 125 meV (*23*), corresponding to a temperature of 1400 K and a density of $3.5 \times 10^{12}\mathrm{cm}^{-2}$. Therefore intrinsic optical phonon scattering can be ignored for the purposes of this study.

We also consider scattering from polar optical phonons within the boron nitride substrate, using methods reported previously (*23, 44, 45*). The scattering rate of an electron of energy $\epsilon_{\mathbf{k}} = \hbar^2 k^2/2m^\star$ with each of the two polar phonon modes of hBN (denoted $\nu = 1, 2$) is given by

$$\begin{aligned}\frac{1}{\tau^{(\nu)}_{e-sph}(k)} &= \frac{2\pi}{\hbar}\sum_{\mathbf{q}}\left|\frac{M^{ss'}_{\nu}(q,\theta_{\mathbf{q}})}{\varepsilon(q,\omega=0,\mu,T)}\right|^2 [(N_\nu + n_F(\epsilon_{\mathbf{k+q}}))\delta\left(\epsilon_{\mathbf{k+q}} - \epsilon_{\mathbf{k}} - \hbar\omega^{(\nu)}_{sph}\right) \\ &\quad +(N_\nu + 1 - n_F(\epsilon_{\mathbf{k+q}}))\delta\left(\epsilon_{\mathbf{k+q}} - \epsilon_{\mathbf{k}} + \hbar\omega^{(\nu)}_{sph}\right)], \qquad \text{(S20)}\end{aligned}$$

where

$$\begin{aligned}|M^{ss'}_{\nu}(q,\theta_{\mathbf{q}})|^2 &= e^2\frac{\hbar\omega^{(\nu)}_{sph}}{2A\varepsilon_0}\left(\frac{1}{\kappa_\nu + 1} - \frac{1}{\kappa_{\nu-1}+1}\right)\frac{e^{-2qd}}{q} \\ &\quad \times\frac{1}{2}\left(1 + ss'\cos(2\theta_{\mathbf{k},\mathbf{k+q}})\right). \qquad \text{(S21)}\end{aligned}$$

Above, A is the area of the bilayer graphene and $d = 3.5$ Å (*46*) is the van der Waals distance between bilayer graphene and hBN and the substrate phonon energies are given by $\hbar\omega^{(\nu=1,2)}_{sph} = 101.6, 195.7$ meV respectively. The zero, intermediate and high frequency dielectric constants $\kappa_{0,1,2} = 5.09, 4.575, 4.10$ respectively. ε refers to the RPA dielectric function defined in Eq. (S26) below and ε_0 is the vacuum permittivity. N_ν and n_F are the Bose-Einstein and Fermi-Dirac distributions respectively. The substrate phonon scattering rate shown in Fig. S4 above is obtained by taking the sum of scattering from the two modes $1/\tau^{(1)}_{e-sph} + 1/\tau^{(2)}_{e-sph}$ and performing the thermal average shown in Eq. (S19).

Figs. S4(a) and (b) show the the scattering rates of electrons with acoustic and the substrate polar phonon modes, respectively, as a function of temperature. At all temperatures relevant to the experiments, the acoustic phonon scattering rate is more than an order of magnitude greater than the hBN substrate phonon scattering rate. Likewise, the resistivity due to acoustic phonon scattering far exceeds greatly exceeds the substrate phonon limited resistivity (Fig. S4c). The insignificance of both intrinsic and substrate optical phonon scattering is easily understood qualitatively because the optical phonon energies of $\sim$ 100 meV ($\sim$ 1200 K) lead to a small population of these modes within the temperature range considered in this work (*23, 44*).

3.3 Impurity scattering

We calculate the impurity scattering time using the standard expression for inverse quasiparticle lifetime due to charged impurities (*24, 35*)

$$\frac{1}{\tau\left(\epsilon_{k,\gamma}\right)} = \pi n_{imp} \int \frac{d^2 k^{'}}{(2\pi)^2} \left| \frac{v_q}{\varepsilon(q, \omega = 0, \mu, T)} \right|^2 \delta(\epsilon_{k,\gamma} - \epsilon_{k^{'},\gamma}) F_{\gamma,\gamma}(\theta_{\mathbf{k},\mathbf{k}-\mathbf{q}}), \tag{S22}$$

where n_{imp} is the charged impurity density, $\mathbf{k}^{'} = \mathbf{k} - \mathbf{q}$,

$$F_{\gamma,\gamma^{'}}(\theta_{\mathbf{k},\mathbf{k}-\mathbf{q}}) = \frac{1}{2}(1 + \mathrm{sign}(\gamma)\mathrm{sign}(\gamma^{'}) \cos(2\theta_{\mathbf{k},\mathbf{k}-\mathbf{q}})), \tag{S23}$$

$\theta_{\mathbf{k},\mathbf{k}-\mathbf{q}}$ is the angle between $\mathbf{k}$ and $\mathbf{k} - \mathbf{q}$, and ε refers to the RPA dielectric function defined in Eq. (S26) below. We set $n_{\mathrm{imp}} = 10^{10}$ cm^{-2}, $\gamma = 1$ above for electrons (-1 is for holes) and perform the thermal average shown in Eq. (S19). We find that the scattering rate is largely independent of carrier density and temperature over the ranges relevant to this experiment (see Fig. S5), and thus may be approximated as a constant value τ_{imp} for the purposes of fitting to experimental data (as is normally done in the literature).

Our Hall measurements in Fig. S2 (upper inset) indicate that the charged impurity density is $n_{\mathrm{imp}} \sim 10^{10}\mathrm{cm}^{-2}$. This value corresponds to an impurity scattering time of $\tau_{imp} \sim 4$ ps (Fig. S5). Therefore, in Fig. 2A of the main text, we use a value of $\tau_{imp} = 8$ ps to calculate

an upper bound for the impurity-limited conductivity at charge neutrality. We use a value of $\tau_{imp} = 0.8$ ps (corresponding to $n_{\text{imp}} \sim 5 \times 10^{10}\text{cm}^{-2}$) to calculate the lower bound, since larger values of n_{imp} are ruled out by the Hall measurements as well as existing literature (*35*).

In fitting the density- and temperature dependent resistivity data, τ_{imp} is kept as a free parameter. We find that the best match to the data is provided by $\tau_{imp} = 5$ ps, matching the value calculated above using the charged impurity density derived from the Hall measurements.

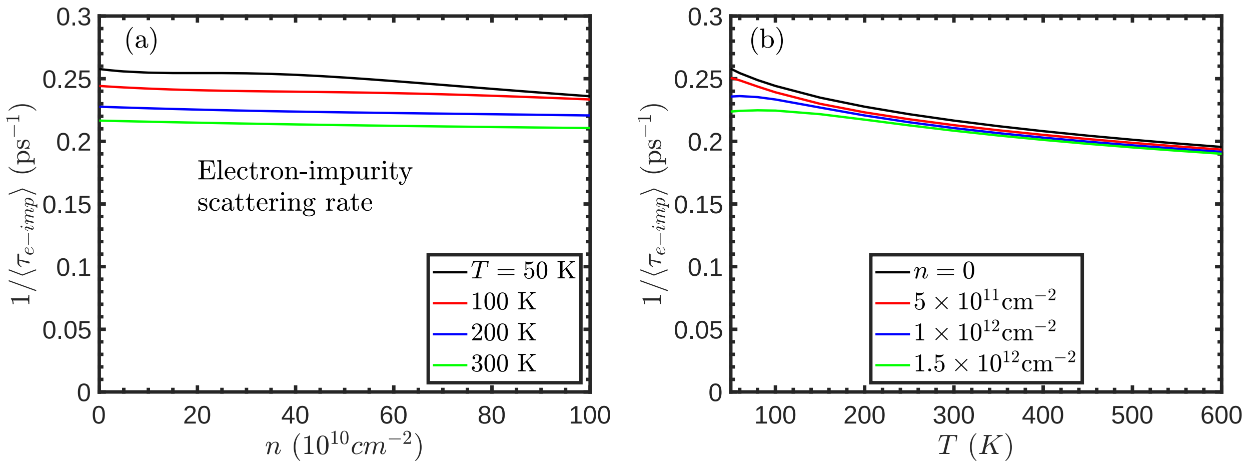

Figure S5: **Electron-impurity scattering.** Electron-impurity scattering rates as a function of (a) density and (b) temperature. The scattering rates change gradually with respect to both quantities.

3.4 Electron-hole scattering

The finite temperature quasiparticle lifetime $\tau\left(\epsilon_{k,\gamma}\right)$ of a quasiparticle at energy $\epsilon_{k,\gamma}$ due to scattering with quasiparticles in the opposite band γ' is given within the Random Phase Ap-

proximation (RPA) by the expression (*47*)

$$\begin{aligned}
&\frac{1}{\tau\left(\epsilon_{k,\gamma}\right)} \\
= \; & -\frac{2}{(2\pi)^2}\sum_{\gamma',\gamma''}\int_{-\infty}^{\infty} d\omega \frac{1-f(\epsilon_{k,\gamma}-\omega)}{1-\exp(-\beta\hbar\omega)}\int_0^{\infty} dq\, q \Big|\frac{v_q}{\varepsilon(q,\omega,\mu,T)}\Big|^2 \mathrm{Im}\chi^{(0)}_{-\gamma,\gamma''}(q,\omega,\mu,T)A_{\gamma,\gamma'}(k,q,\omega) \\
& +\frac{2}{(2\pi)^2}\sum_{\gamma',\gamma''}\int_{-\infty}^{\infty} d\omega \frac{f(\epsilon_{k,\gamma}-\omega)}{1-\exp(\beta\hbar\omega)}\int_0^{\infty} dq\, q \Big|\frac{v_q}{\varepsilon(q,\omega,\mu,T)}\Big|^2 \mathrm{Im}\chi^{(0)}_{-\gamma,\gamma''}(q,\omega,\mu,T)A_{\gamma,\gamma'}(k,q,\omega),
\end{aligned} \tag{S24}$$

where

$$v_q = \frac{2\pi e^2}{\kappa q} \tag{S25}$$

is the bare Coulomb interaction and $\kappa = 3.5$ is the dielectric constant for bilayer graphene mounted on hBN. μ denotes the chemical potential and T denotes temperature. $\varepsilon(q,\omega,\mu,T)$ is the RPA dielectric function (not to be confused with the energy $\epsilon_{k,\lambda}$) given by

$$\varepsilon(q,\omega,\mu,T) = 1 - \chi^{(0)}(q,\omega,\mu,T)v_q, \tag{S26}$$

and

$$\chi^{(0)}(q,\omega,\mu,T) = \sum_{\nu,\nu'}\chi^{(0)}_{\nu,\nu'} \tag{S27}$$

is the Lindhard polarizability function and the summation indices run over the carrier species e and h, with components

$$\chi^{(0)}_{\nu,\nu'} = g\lim_{\eta\to 0}\int \frac{d^2k'}{(2\pi)^2}\frac{f(\epsilon_{\mathbf{k}',\nu}) - f(\epsilon_{\mathbf{k}'+\mathbf{q},\nu'})}{\hbar\omega + \epsilon_{\mathbf{k}',\nu} - \epsilon_{\mathbf{k}'+\mathbf{q},\nu'} + i\eta}F_{\nu,\nu'}(\theta_{\mathbf{k},\mathbf{k}-\mathbf{q}}), \tag{S28}$$

with $g = 4$ being the degeneracy factor for bilayer graphene. The first subscript of $\mathrm{Im}\chi^{(0)}_{\nu,\nu'}$ is set to $-\gamma$ in Eq. (S24) for collisions between carriers from different bands. (To get the scattering rate for carriers in the same band, simply set ν to γ instead.)

The $A_{\gamma,\gamma'}(k,q,\omega)$ term is given by

$$A_{\gamma,\gamma'}(k,q,\omega) \equiv \int_0^{2\pi} d\theta_{\mathbf{q}}\delta(\epsilon_{k,\gamma} - \epsilon_{\mathbf{k}-\mathbf{q},\gamma'} - \hbar\omega) \times F_{\gamma,\gamma'}(\theta_{\mathbf{k},\mathbf{k}-\mathbf{q}}), \tag{S29}$$

where

$$F_{\gamma,\gamma'}(\theta_{\mathbf{k},\mathbf{k}-\mathbf{q}}) = \frac{1}{2}(1 + \mathrm{sign}(\gamma)\mathrm{sign}(\gamma')\cos(2\theta_{\mathbf{k},\mathbf{k}-\mathbf{q}})) \tag{S30}$$

and $\theta_{\mathbf{k},\mathbf{k}-\mathbf{q}}$ is the angle between $\mathbf{k}$ and $\mathbf{k}-\mathbf{q}$. We substitute the above $\tau(\epsilon_{k,\gamma})$ into the thermal average Eq. (S19) for $\gamma = 1$ and display our results in black in Fig. S3. At charge neutrality $\mu = 0$, one can show by substitution of Eq. (S24) for both $\gamma = \pm 1$ into Eq. (S19) that $1/\langle\tau\rangle$ is linearly proportional to temperature in both cases, leading to $1/\tau_0 \equiv 1/\langle\tau_e(\mu = 0)\rangle + 1/\langle\tau_h(\mu = 0)\rangle = \alpha_0 k_B T/\hbar$, with $\alpha_0 \sim 0.2$. This justifies the form $1/\tau_0 = \alpha_0 k_B T/\hbar$ used in the main text with α_0 as a constant fit parameter. Fig. S3 also shows an exponential drop of $1/\tau_{eh}$ away from neutrality, consistent with the equation $\tau_{eh} = (n_e + n_h)/(n_h) \times \tau_0$ used in the main text. For completeness, we show also results for the thermally averaged electron-electron scattering rate in red in Fig. S3.

4 Extraction of relaxation times from experiment

4.1 Details of the fitting procedure

Here we detail the procedure used to extract the relaxation rates from experiment. The hydrodynamic conductivity given by the sum of σ_{c} and σ_{dis} in Eqs. (S12) and (S13) contains three undetermined parameters α_0 and $\tau_{\mathrm{e/h,dis}}$. We first determine the electron-hole scattering parameter α_0 by examining the temperature-independent conductivity at neutrality shown in Fig 2A of the main text. This data is shown in greater detail in figure in Fig. S6A. As can be seen, devices 2 and 3 show near-identical conductivity between 100 K and 300 K, while device 1 shows slightly lower conductivity below 200 K. Fig. Fig. S6B shows the measured resistivity across the charge neutrality point for the same samples. The width (FWHM) of the resistivity peak provides a good estimate of the device disorder (*48,49*). All three devices show FWHM in the range of 10^{11} cm^{-2}, in good agreement with the detailed Hall effect data shown for device 3 in Fig. S2. However, it is clear that device 1 has a slightly larger FWHM, indicating somewhat larger disorder. In addition, sample 1 shows a small extra peak in resistance at small negative density. This may be due to a superlattice moire pattern that modifies the low-energy bandstructure. For these reasons, the low-T data for device 1 may not precisely reflect the intrinsic behavior of bilayer graphene (at higher temperatures these effects are less important).

We also note that the apparent anomalous behavior (i.e. slight rise in conductivity) of device 2 below 100 K is likely an experimental artifact. Its behavior was measured using a fixed grid of gate voltage points, which were not fine enough to precisely capture the exact point of charge neutrality when the resistivity peak becomes extremely narrow at low T. Device 3 was measured by simultaneously sweeping top and bottom gates to vary the net density more finely, and was able to precisely follow the charge neutrality point even at low temperature. Based on the above reasoning, we determine the value of α_0 from the observed conductivity of devices 2 and 3

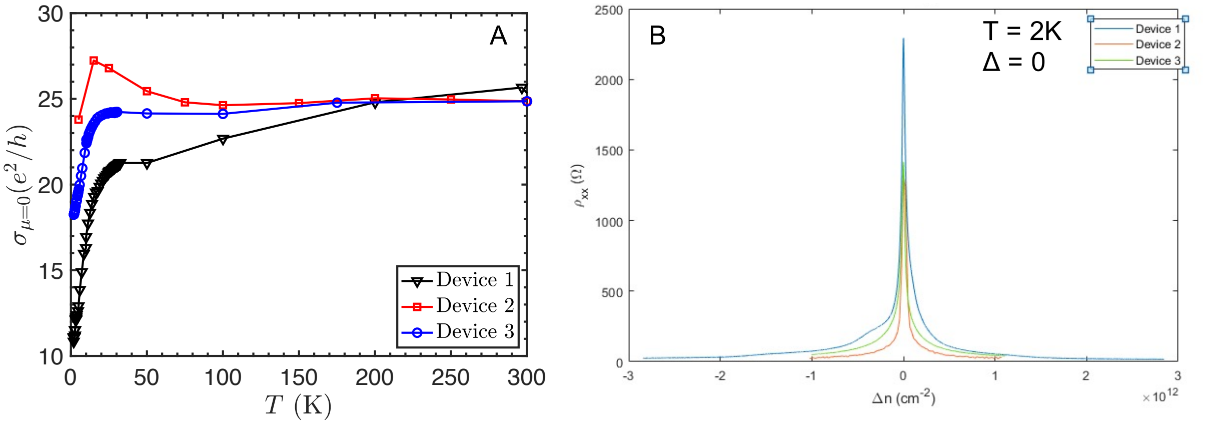

Figure S6: **Sample variability.** (A) Measured conductivity (solid points) at $\Delta, \mu = 0$ for devices 1,2, and 3, from 0 K to 300 K. The lines are a guide to the eye only. (B) Measured resistivity vs. net carrier density (Δn) at T=2K for the same three samples. The cleanest samples with the narrowest peaks in $\rho_{xx}(\Delta n)$ (device 2 and 3) show the least variability.

between 100 K and 300 K. This gives a value of $\alpha_0 = 0.225 \pm 0.002$.

We next measured $\sigma(\mu)$ for sample 3 at fixed temperatures of 50, 100, 175, and 300 K. This data is shown as discrete points in Figure 2B of the main text and Fig. S7. A least squares fit of hydrodynamic conductivity to the experimental conductivity is then performed at each temperature, with the two $\tau^{-1}_{\mathrm{e/h,dis}}$ as fit parameters and α_0 fixed at 0.225. The resulting values obtained for $\tau^{-1}_{\mathrm{e/h,dis}}$ are shown in the left half of Table 2 below, and the resulting theoretical conductivities using these extracted parameters are displayed in Fig. S7A above. For comparison, we repeat in Fig. S7B the same fitting procedure for $\tau^{-1}_{\mathrm{e/h,dis}}$ using a phenomenological Matthiessen's rule

$$\sigma = \frac{n_e e^2}{m^*}\left(\frac{1}{\tau_{eh}} + \frac{1}{\tau_{e,dis}}\right)^{-1} + \frac{n_h e^2}{m^*}\left(\frac{1}{\tau_{he}} + \frac{1}{\tau_{h,dis}}\right)^{-1}, \tag{S31}$$

where $\tau_{eh} = \tau_0(n_e + n_h)/(2n_h)$, $\tau_{he} = \tau_0(n_e + n_h)/(2n_e)$ and $\tau_0 = \hbar/(\alpha_0 k_B T)$. It is clear that Matthiesen's rule is unable to produce good agreement with experiment. Only hydrodynamic conductivity is able to reproduce the experimental data. Note that because the hydrodynamic Eqs. (S12) and (S13) agree with Matthiessen's rule at charge neutrality (up to terms

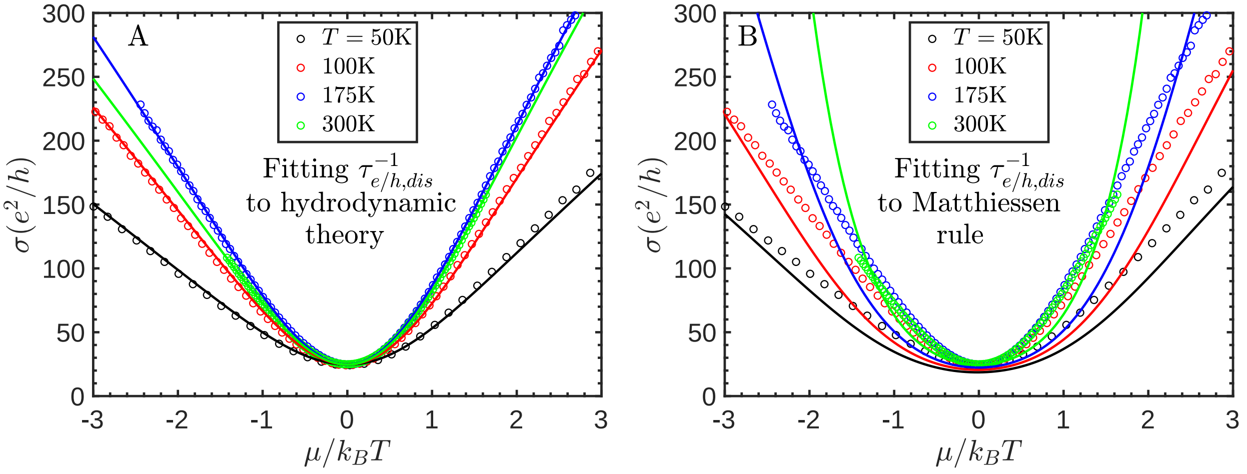

Figure S7: **Additional data for the gapless conductivity.** (A) Comparison of theoretical conductivity (solid lines) using hydrodynamic theory with experimental data (symbols). (B) The same using Matthiessen's rule. In both cases, we use the value $\alpha_0 = 0.225$ obtained from fitting to σ_0 in Fig. 2A of main text, and for $\tau^{-1}_{\mathrm{e/h,dis}}$ we use the values obtained by least square fitting to the data at each temperature. The values obtained from fitting using hydrodynamic theory are displayed in Table 2 below.

of $O(\tau_0/\tau_{e/h,dis})$) where $\tau^{-1}_{eh/he} \gg \tau^{-1}_{e/h,dis}$, and also in the opposite high density regime where $\tau^{-1}_{eh/he} \ll \tau^{-1}_{e/h,dis}$, it is possible to obtain agreement with experiment at charge neutrality *or* high density, but not across the the entire range.

In Fig. 3B of the main text, we perform a least squares fit of $\alpha^{(e/h)}_{ac} k_B T/\hbar + \tau^{-1}_{imp}$ to the $\tau^{-1}_{\mathrm{e/h,dis}}$ values obtained above, using $\alpha^{(e/h)}_{ac}$ and τ^{-1}_{imp} as fit parameters. The resulting values are displayed in the right half of Table 2 below. Note that τ_{imp} is the same for electrons and holes unlike $\alpha^{(e/h)}_{ac}$ due to the difference in effective mass between electrons and holes (*27*).

4.2 Ruling out ballistic transport

Given that our samples are ultraclean, it is a valid concern as to whether electrons are simply traveling across the sample ballistically without undergoing any current-relaxing scattering events. Indeed, Nam et al (*14*) measured a negative bend resistance indicating ballistic transport

Table 2: **List of all extracted fitting parameters.** (Left) Table of dissipative momentum relaxation rates extracted from experiment at each temperature from the fitted curves shown in Fig. S7A above. (Right) Table of the phonon, impurity and electron-hole scattering parameters obtained from Fig. 3B of the main text by fitting the extracted dissipative momentum relaxation rates via $\tau^{-1}_{\mathrm{e/h,dis}} = \alpha^{(e/h)}_{ac} k_B T/\hbar + \tau^{-1}_{imp}$.

T (K)	$\tau^{-1}_{e,dis}$ (ps^{-1})	$\tau^{-1}_{h,dis}$ (ps^{-1})
50	0.442 ± 0.002	0.519 ± 0.004
100	0.567 ± 0.002	0.685 ± 0.003
175	0.790 ± 0.002	0.961 ± 0.003
300	1.408 ± 0.002	1.869 ± 0.004

$\alpha^{(e)}_{ac}$	0.030 ± 0.008
$\alpha^{(h)}_{ac}$	0.041 ± 0.008
τ^{-1}_{imp}	0.2 ± 0.2 ps^{-1}
α_0	0.225 ± 0.002

for Fermi energies E_F above $k_B T$ in their suspended samples that were $\sim 2\mu\mathrm{m}$ in both width and length. At $T = 50$ K (the lowest temperature used for analysis of hydrodynamic transport) this corresponds to a density of about $3 \times 10^{11}\mathrm{cm}^{-2}$ and at at $T = 300$ K this corresponds to a density of about $2 \times 10^{12}\mathrm{cm}^{-2}$.

The electron (hole) mean free path l at carrier density $|n|$ can be extracted from the measured conductivity according to $\sigma = 2e^2/h\sqrt{\pi|n|}l$. Since the low-temperature conductivity increases linearly with $|n|$, the mean free path increases roughly as $\sqrt{|n|}$ and is greatest at high carrier density. At 50 K and at density of $|n| = 3 \times 10^{11}\mathrm{cm}^2$, the measured conductivity of $150\frac{2e^2}{h}$ gives a mean free path of roughly $0.75\mu\mathrm{m}$. This is smaller than the smallest dimension of any of the samples studied here (see Table 1 of Section 1 above). Likewise, at 300 K and density of $|n| = 1 \times 10^{12}\mathrm{cm}^2$ (the highest density studied here), the conductivity is also roughly $150\frac{2e^2}{h}$, corresponding to a mean free path of $0.4\mu\mathrm{m}$. We note that at charge neutrality, $|n|$ is not given by the net charge but the total density of electrons and holes due to thermal excitation and static charge disorder. At 50 K this value is roughly $|n| = 10^{11}\mathrm{cm}^2$, leading to a mean free path of $0.2\mu\mathrm{m}$. Therefore we can conclude that the samples in this study are diffusive in the regime in which hydrodynamic transport was analyzed.

4.3 Determination of the hydrodynamic transport regime

While much experimental effort has been dedicated to the study of the electronic viscosity in the hydrodynamic regime, comparatively little has been dedicated to the influence of hydrodynamics on electrical transport, presumably because carrier-carrier scattering conserves total carrier momentum and thus cannot by itself yield a finite electrical resistance in general (see below for the exception). To put it more clearly, carrier-carrier scattering amounts to an internal force within the gas of charge carriers that cannot prevent its center-of-mass from accelerating to infinite velocity under a non-zero net force caused by an external electric field. The current induced by an external field in the presence of only carrier-carrier scattering is thus expected to be infinite.

An exception to this rule occurs in the case where electrons and holes co-exist in equal number, because the net force exerted on the gas of carriers (i.e. the combined gas of electrons and holes) by the external electric field is zero. In this special case, the internal forces within the combined gas due to electron-hole friction (i.e. electron-hole scattering) are sufficient to prevent the charges from accelerating to infinite speed, thus leading to finite electrical resistance. In detail, upon switching on an external electric field, the individual electron and hole gases initially accelerate in opposite directions due to the field, but since the electron-hole frictional force increases with the difference in their relative velocity, this acceleration continues only up to the point where each gas experiences a frictional force that cancels the external field. The velocity of each gas then remains at this constant value, yielding a finite current and conductivity. The hydrodynamic regime is thus expected to have a direct impact on electrical transport whenever electrons and holes are present in equal density, a situation that is naturally realised in bilayer graphene at the charge neutrality point.

Nam *et al.* (*14*) were the first to experimentally realise this situation using ultraclean suspended bilayer graphene. This work found a novel collapse of conductivity as a function of the

ratio of Fermi energy to temperature over a range of finite but low charge densities near charge neutrality and at various temperatures, which the authors attributed solely to electron-hole scattering. This conclusion was however problematic because as explained above, electron-hole collisions alone can only yield a well-defined resistance at precise charge neutrality. Subsequent theoretical work (*12*) showed that the conductivity collapse was in fact due to an interplay of electron-hole scattering and the next fastest scattering process- that of scattering between electrons and in-plane acoustic phonons.

Fig. S3 above gives a sense of where the electron-hole dominant hydrodynamic regime lies. It shows that a low-density window in which the electron-hole scattering rate τ_{eh}^{-1} dominates at all temperatures above 50K, with the size of this widow increasing with temperature.

The electron-impurity scattering rate in monolayer graphene may be estimated by $\tau_{\rm imp}^{-1} \approx 68 \times n_{\rm imp}[10^9 {\rm cm}^{-2}]/T[K]$ THz (*50*) assuming encapsulation in hexagonal boron nitride with a dielectric constant of 4. According to a recent careful study of scattering times in ultraclean monolayer graphene (*13*), the electron-hole scattering rate is given by $\tau_{eh}^{-1} \approx 0.20 k_B T/\hbar$ and $n_{\rm imp} = 2.1 \times 10^9 {\rm cm}^{-2}$ in the cleanest samples. Given the above estimates, electron-impurity and electron-hole scattering rates are equal at ~ 70 K. Achieving equality at 10 K would then require $n_{\rm imp} \approx 4 \times 10^7 {\rm cm}^{-2}$. On the other hand, our calculation for bilayer graphene using the expression given above in Eq. (S29) shows that a realistic impurity concentration of $n_{\rm imp} = 10^{10} {\rm cm}^{-2}$ corresponds to $\tau_{\rm imp}^{-1} = 0.25$ THz. Given $\tau_{eh}^{-1} \approx 0.15 k_B T/\hbar$ (*12*) in bilayer graphene, the electron-impurity and electron-hole scattering rates are equal at $\sim 10K$.

Using the model developed in this paper, we illuminate the relationship between electron-hole scattering and disorder. As in the main text, we use Eqs. (S12) and (S13) to calculate the reduction in conductivity due to electron-hole scattering $\Delta\sigma = \sigma_{ac+i} - \sigma_{\rm total}$, where σ_{ac+i} is the conductivity incorporating only acoustic phonons and impurities, and $\sigma_{\rm total}$ is the total conductivity.

Here we have kept all electron-hole and phonon scattering parameters consistent with those used in Fig. 5 of the main text and varied only the impurity momentum relaxation time $\tau_{\rm imp}$, which takes the values 40, 4, 0.4 and 0.04 ps respectively for the aforementioned values of $n_{\rm imp}$. The window for electron-hole scattering limited transport is greatly diminished across all temperatures with higher $n_{\rm imp}$ as impurity-limited transport dominates. This result highlights the importance of ultra-clean bilayer graphene for hydrodynamic transport, and the degree to which hexagonal boron nitride encapsulation is needed to provide such a platform. The electron-hole limited regime vanishes for impurity concentrations $\gtrsim 10^{11}$cm^{-2}, explaining why this regime was never observed in early samples possessing higher levels of disorder.

5 Bilayer graphene with a bandgap

Figure S2A shows the schematic bandstructure of bilayer graphene, which can be approximated as two hyperbolic bands, with dispersion $\epsilon_{\pm}(k) = \pm\sqrt{(\hbar^2k^2/(2m^*))^2 + (\Delta/2)^2}$, where $\pm$ denote the conduction and valence bands, $\hbar$ is Planck's constant, k the wave vector, and m^* the effective mass. Three relevant energy scales are shown: the field-tunable bandgap Δ, and the chemical potential μ and thermal energy k$_B$T, which determine the density of thermally excited electrons and holes (n_e and n_h). This dispersion is known to be valid for electrons of energy less than 0.4 eV (*38, 51*), corresponding to density $n = 10^{13}$ cm^{-2} and temperature 4600 K, making it applicable to our experiment that is restricted to $n \lesssim 10^{12}$ cm^{-2}.

5.1 Device characterization and experimental control of carrier density and bandgap

The devices were measured by biasing a small current (10 - 100 nA) between two outer leads, then measuring the voltages between the center longitudinal and transverse leads V_{xx} and V_{xy}, respectively, as shown in Fig. S1. The currents $\sim$ 10 nA ensure we are well within the range

in which electrons may be considered to be in thermal equilibrium with the lattice even in the presence of strong electron-hole scattering (*19*). We calculate the resistances by dividing the voltages by the current bias, using the results to derive the conductivity via the tensor relation:

$$\sigma = \frac{LR_{xx}}{W(R_{xx}^2 + R_{xy}^2)} \tag{S32}$$

Where L and W are device dimensions. The measured charge neutrality resistances of $\approx 10^5$ Ω in a current bias scheme show the system to be free from conducting defects such as strained soliton networks (*52*), consistent across devices in this work. The dual gate devices allow us to independently control density and band gap in bilayer graphene using the top and bottom gates V_{TG} and V_{BG}, respectively. The measured low-T resistance of one device as a function of applied top and bottom gate voltages (V_{TG} and V_{BG}) is shown in Fig. S2C. The resistance peak along the diagonal tracks the charge neutrality point ($\mu = 0$), with the emergence of a bandgap with displacement field appearing as increasing resistance toward top left and bottom right. These data, together with low-temperature Hall effect measurements (Fig. S2D insets), allow determination of the individual top and bottom gate capacitances (see supplementary material). We introduce two parameters V_{eff} and Δ_{ext}, which tune μ and Δ with one-to-one correspondence, respectively. We calculate the electrostatic potential V_{eff} at a given constant displacement field D (and therefore band gap Δ) as:

$$V_{\text{eff}} = \frac{(V_{\mathrm{TG}} - V_{\mathrm{TG(CNP)}}) - sV_{\mathrm{BG}}}{\sqrt{1+s^2}}. \tag{S33}$$

Here $s = -C_{\mathrm{TG}}/C_{\mathrm{BG}}$ is the negative ratio of the top and bottom gate capacitances and can be extracted from the slope of the charge neutrality point (CNP) (where $\Delta n = 0$) when plotting R_{xx} against V_{TG} and V_{BG}. $V_{\mathrm{TG(CNP)}}$ is the CNP offset at $V_{\mathrm{BG}} = 0$. We also independently tune Δ with Δ_{ext}, which is calculated as:

$$\Delta_{\mathrm{ext}} = eDc_0 = \frac{ec_0}{2}\left[\frac{\epsilon_{\mathrm{TG}}(V_{\mathrm{TG}} - V_{\mathrm{TG0}})}{t_{\mathrm{TG}}} - \frac{\epsilon_{\mathrm{BG}}(V_{\mathrm{BG}} - V_{\mathrm{BG0}})}{t_{\mathrm{BG}}}\right], \tag{S34}$$

where $\Delta_{\mathrm{ext}} = 0$ at V_{TG0}, V_{BG0}, e is the electron charge, $\epsilon_{\mathrm{TG(BG)}}$ is the top (bottom) boron nitride dielectric constant, $t_{\mathrm{TG(BG)}}$ is the top (bottom) boron nitride thickness, and c_0 is the BLG interlayer spacing. The relation between Δ_{ext} and Δ is monotonic but not straightforward, and theoretical studies of this relation vary depending on the model and details considered. In the ranges considered in this paper, we can approximate the relation between the two as linear, $\Delta_{\mathrm{ext}} \approx 2.6\Delta$ as determined experimentally from Arrhenius fittings, in good agreement with tight-binding models (*37*). We note that in the calculation of V_{eff} with various Δ_{ext} the charge neutrality points do not align unless $s = -1$, and we've renormalized V_{eff} to $V_{\mathrm{eff}} - V_{\mathrm{eff(CNP)}}$ for ease of comparison.

Fig. S2D show the measured conductivity as a function of V_{eff} for $\Delta_{\mathrm{ext}} = 0$ and 150 meV, at temperatures from 5K to 300K. Away from charge neutrality, both plots show metallic behavior. At charge neutrality, conductivity decreases upon cooling for $\Delta_{\mathrm{ext}} = 150$ meV, as expected from the opening of a band gap. Strikingly, for $\Delta_{\mathrm{ext}} = 0$, the charge-neutral conductivity is large ($\sim 20\frac{e^2}{h}$) and temperature-independent. The insets of Fig. S2D show Δn as a function of V_{eff} at $T = 2$K for $\Delta_{\mathrm{ext}} = 0$ and $\Delta_{\mathrm{ext}} = 150$ meV, as determined from Hall effect measurements. The linear variation with V_{eff} to below $\approx 10^{11}\mathrm{cm}^{-2}$ sets an upper bound on charge disorder of $\sim 3 \times 10^{10}\mathrm{cm}^{-2}$, confirming that the devices are in the low-disorder limit. The corresponding plot for $\Delta_{\mathrm{ext}} = 150$ meV shows ~ 50 meV separation between the electron and hole branches, reflecting the induced band gap. At low temperatures such as $T = 2$K, carrier freeze out allow us to neglect minority carriers and use the single carrier model. We calculate the single carrier charge density Δn from the Hall coefficient R_{H} per the relation $R_{\mathrm{H}} = -1/\Delta ne$, where e is the electron charge and R_{H} the slope of the linear relation between R_{xy} vs. B. A typical R_{xy} vs. B plot is shown in Fig. S8.

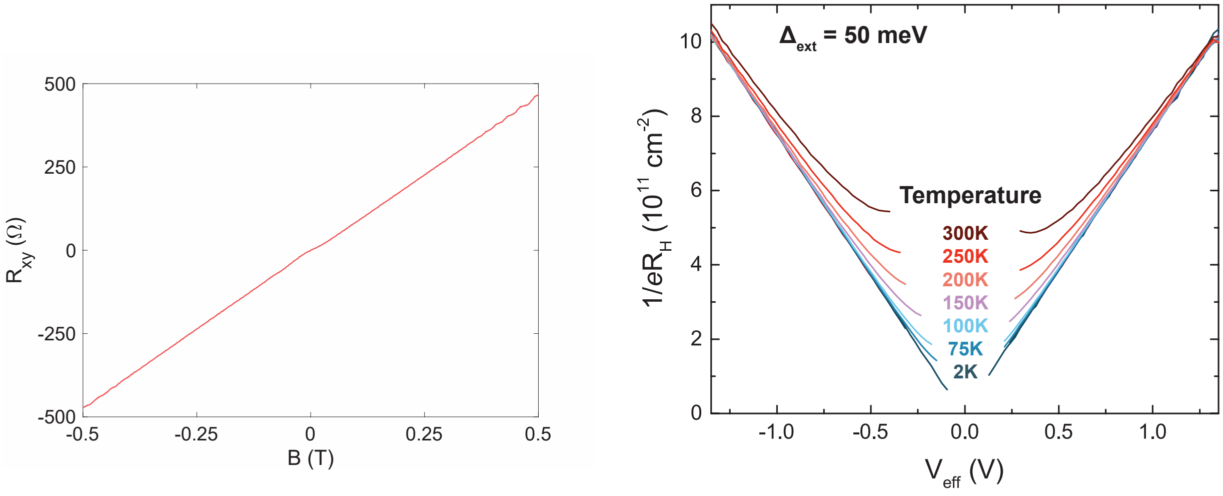

Figure S8: **Measuring the charge density.** (a) A typical plot of R_{xy} vs B, from which the Hall coefficient R_{H} and charge density Δn can be extracted. (b) Plotted $1/eR_{\text{H}}$ as a function of V_{eff}. At low temperatures, the transport is single carrier dominated and the lines show the gate dependence of Δn. At higher temperatures, however, both electrons and holes exist near the charge neutrality point, and the single carrier transport model breaks down, resulting in non-linearity.

At higher temperatures, carrier thermalization breaks down the single carrier picture near the charge neutrality point. As shown in Fig. S8, $1/eR_{\text{H}}$ (the calculated single carrier charge density) deviates from a linear dependence upon V_{eff} near the charge neutrality point at higher temperatures. This is due to the thermal population of minority carriers, even at considerably large band gaps. The single carrier model is therefore insufficient at higher temperatures, the region of most interest for hydrodynamic transport. Instead, we turn to the ambipolar transport model to extract the Hall density. The conductivity for such a system is given by:

$$\sigma = e(n_e u_e + n_h u_h) \tag{S35}$$

where the $n_e(h)$ and $u_e(h)$ are the electron (hole) density and mobility, respectively, and the mobility can be understood as $u = \frac{e\langle\tau\rangle}{m}$, where $\langle\tau\rangle$ is the carrier relaxation time. Here we use u instead of the standard μ for mobility as to avoid confusion with the chemical potential. The

Hall coefficient for the ambipolar system also becomes:

$$R_{\mathrm{H}} = \frac{n_h {u_h}^2 - n_e {u_e}^2}{e(n_e u_e + n_h u_h)^2} \tag{S36}$$

By assuming the carrier mobilities to be dominated by electron-hole scattering, we can then calculate the mobilities per:

$$u_e = u_0 \frac{n_e + n_h}{n_h},\ u_h = u_0 \frac{n_e + n_h}{n_e} \tag{S37}$$

Where $u_0 = \frac{e\langle\tau\rangle_0}{m}$ is the carrier mobility at charge neutrality, $\langle\tau\rangle_0$ is calculated below. We can then use the above equations to calculate the electron and hole densities from the measured conductivity and Hall coefficient, provided that $\Delta n = n_e - n_h$ remains consistent with the single carrier Hall density measured at $T = 2$K. We note that while this holds for the gapless case due to the parabolic approximation, it is an extremely rough approximation at large Δ_{ext}, due to quantum capacitance. Nonertheless, this allows us to calculate the minority carrier concentration without the assumption of a band structure. The resulting equation is then:

$$R_{\mathrm{H}} = \frac{(n_e - \Delta n)^3 - {n_e}^3}{e(n_e^2 + (n_e - \Delta n)^2)^2} \tag{S38}$$

From which we numerically calculate the carrier concentrations using the experimentally extracted R_{H} at all temperatures and Δn at $T = 2$K The extracted carrier concentrations for varying temperatures are shown in Fig. S9 for differing Δ_{ext}. Even with a sizable band gap, there is sufficient thermal population of minority carriers near the charge neutrality point at higher temperatures, bringing about hydrodynamic transport.

A key assumption of the ambipolar Hall model requires $\Delta n = n_e - n_h$ at a given V_{eff} to remain constant with temperature. While this assumption is valid for $\Delta = 0$, it does not hold in the case of a finite band gap. A more involved calculation can be done, assuming a band structure. In this section we present how we numerically calculated the chemical potential u

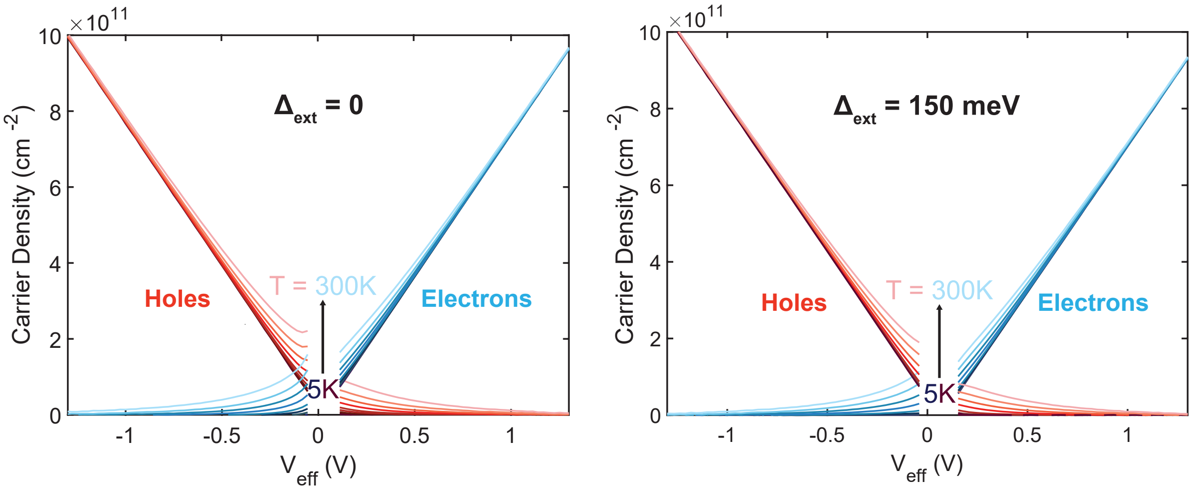

Figure S9: **Measuring the electron and hole densities.** The extracted electron and hole densities for $\Delta_{\text{ext}} = 0$ meV (left) and $\Delta_{\text{ext}} = 150$ meV (right), for $T = 5 \rightarrow 300$K. The thermalization of carriers is shown to increase both minority and majority carriers near the charge neutrality point.

from V_{eff} with a hyperbolic band gap. The total capacitance of the system can be written as

$$C = e\frac{\partial n}{\partial V_{\text{eff}}} = \frac{C_{\text{eff}} C_q}{C_{\text{eff}} + C_q} \tag{S39}$$

where

$$\frac{\partial n}{\partial V_{\text{eff}}} = \frac{\partial n}{\partial \mu}\frac{\partial \mu}{\partial V_{\text{eff}}} = \frac{C_q}{e^2}\frac{\partial \mu}{\partial V_{\text{eff}}} \tag{S40}$$

giving the relation between V_{eff} and μ as

$$eV_{\text{eff}} = \mu + \frac{1}{C_{\text{eff}}}\int_0^{\mu} C_q(\mu)\partial\mu \tag{S41}$$

C_{eff} is the effective geometric capacitance as a function of V_{eff}, extracted from Hall measurements. C_q is the quantum capacitance, calculated from the density of states and derivative of the Fermi-Dirac distribution:

$$C_q = \frac{e^2}{kT}\left[\int_{-\infty}^{-\Delta/2} g(E,\Delta)\frac{\exp\left(\frac{\mu-E}{kT}\right)}{\left(1+\exp\left(\frac{\mu-E}{kT}\right)\right)^2}dE + \int_{\Delta/2}^{\infty} g(E,\Delta)\frac{\exp\left(\frac{E-\mu}{kT}\right)}{\left(1+\exp\left(\frac{E-\mu}{kT}\right)\right)^2}dE\right] \tag{S42}$$

The density of states $g(E,\Delta)$ is dependent on the bandgap Δ and given as:

$$g(E,\Delta) = \frac{2m^*}{\pi\hbar^2}\frac{E}{\sqrt{E^2 - (\frac{\Delta}{2})^2}} \tag{S43}$$

Where we approximate the effective mass m^* as $0.033m_e$. Combining Equations S41, S42, and S43, we can then solve numerically for μ given V_{eff}. We plot in Fig. S10 the calculated μ as a function of V_{eff} for $\Delta_{\text{ext}} = 134$ meV, considering temperatures $T = 100, 175, 300$ K. At high temperatures, μ is approximately linear with V_{eff} due to thermalization of carriers.

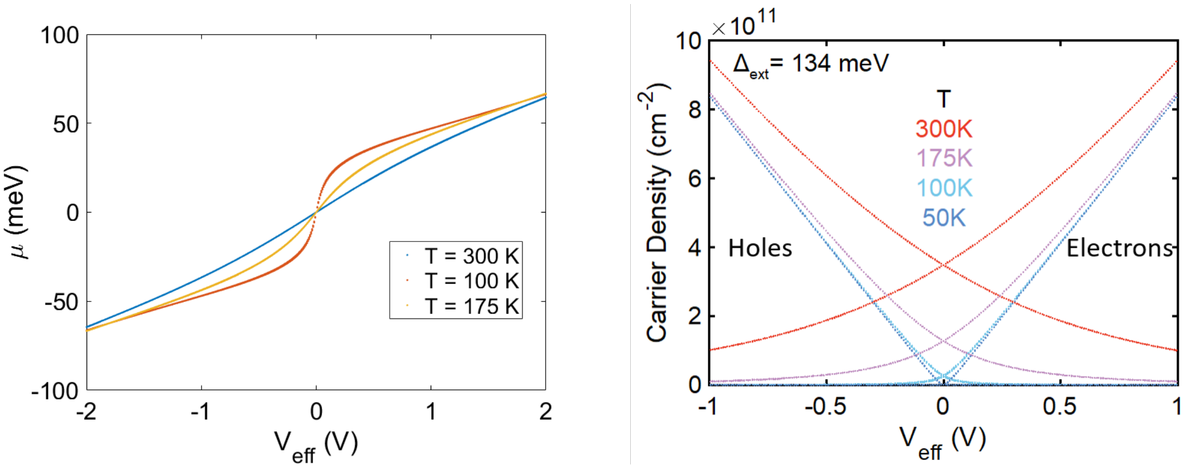

Figure S10: **Calculated chemical potential and carrier density**. (a) Calculated chemical potential plotted against V_{eff} for $\Delta_{ext} = 134$ meV for three distinct temperatures $T = 100, 175, 300$ K. (b) Calculated carrier density as a function of V_{eff} for $\Delta_{\text{ext}} = 134$ meV at temperatures $T = 50, 100, 175, 300$ K.

From μ we can then calculate the carrier densities. Fig. S10 plots the calculated carrier densities as a function of V_{eff} for $\Delta_{\text{ext}} = 134$ meV, temperatures $T = 50, 100, 175, 300$ K. Compared with the experimentally determined carrier densities in Fig. S9, the calculated carrier densities are higher at each V_{eff} when compared to the gapless case. We can then calculate the charge density and check the breakdown of the initial assumption of $\Delta n = n_e - n_h$ and its temperature dependence. Fig. S11 shows that taking quantum capacitance into account, the charge density indeed varies with increasing temperature, as expected due to thermal excitation.

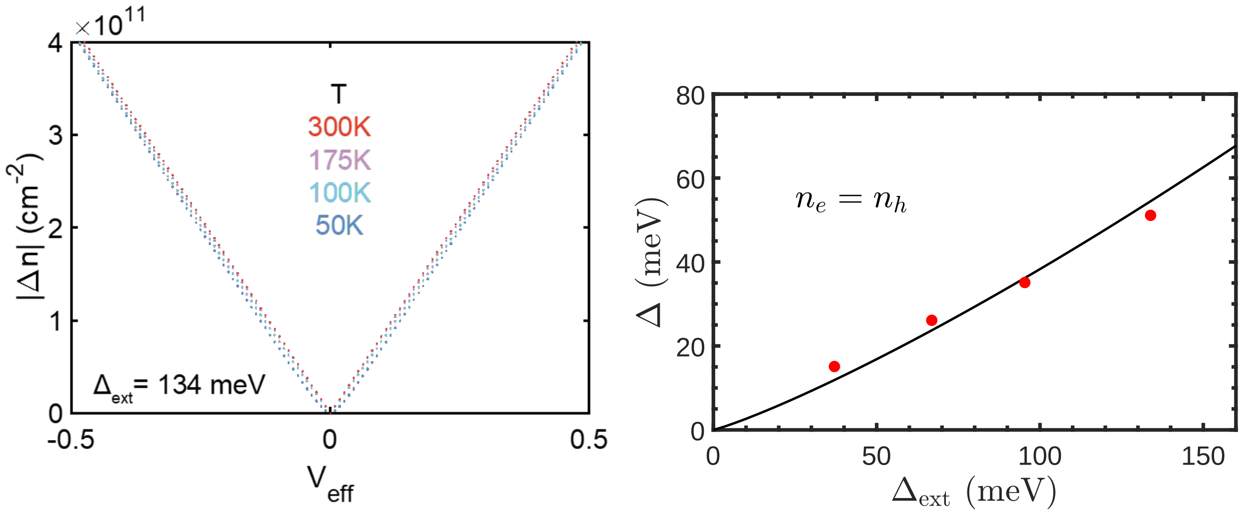

Figure S11: **Calculated charge density and band gaps.** (a) Calculated charge density as a function of $V_{\rm eff}$ for $\Delta_{\rm ext} = 134$ meV at temperatures $T = 50, 100, 175, 300$ K. (b) Self-consistent calculation of the gap size Δ as a function of the external bias strength $\Delta_{\rm ext}$ at charge neutrality where $n_e = n_h$. We plot the experimental band gaps extracted per the Arrhenius relationship alongside in red.

5.2 Self-consistent determination of gap from displacement field

When a transverse displacement field D is applied across bilayer graphene, a band gap of size Δ opens up and the resulting dispersion is approximately hyperbolic, given by (*37*)

$$\epsilon_{\pm}^{(hyp)}(k) = \pm\sqrt{\left(\frac{\hbar^2 k^2}{2m^*}\right)^2 + \left(\frac{\Delta}{2}\right)^2}. \tag{S44}$$

In this work, we determine the size of the gap for a given displacement field D and density $n = n_e - n_h$ self-consistently using the method of Ref. (*37*). This involves evaluating the potential drop across the two graphene layers $\Delta_{\rm ext} = ec_0 D$, where $c_0 = 3.35$ Angstrom is the interlayer separation, and determining Δ numerically in the equation

$$\Delta(n) = \Delta_{\rm ext}\left[1 - \frac{\Lambda}{2}\log\left(\frac{|n|}{2n_\perp} + \frac{1}{2}\sqrt{\left(\frac{n}{n_\perp}\right)^2 + \left(\frac{\Delta}{2\gamma_1}\right)^2}\right)\right]^{-1}, \tag{S45}$$

where $n_\perp = 1.1 \times 10^{13}\text{cm}^{-2}$ is a characteristic density scale. Solving this equation self-

consistently at charge neutrality for Δ reveals the almost perfectly linear relationship between Δ and Δ_{ext} in Fig. S11 below. Experimentally, the relation between Δ_{ext} and Δ is extracted via a linear fit, yielding the $\Delta_{\text{ext}} \approx 2.6\Delta$ relation used in the main text.

5.3 Modification of scattering times in the presence of a bandgap

Within the relaxation time approximation, the non-equilibrium electron distribution $g(\vec{k})$ in the presence of a driving field $\vec{E}$ is

$$g(\vec{k}) = f(\epsilon_k) - e\vec{E} \cdot \vec{v}(\epsilon_{\vec{k}})\tau(\epsilon_k) \left(-\frac{\partial f(\epsilon_k)}{\partial \epsilon_k}\right), \quad \text{(S46)}$$

where $\tau(\epsilon_k)$ is the transport scattering or momentum relaxation time for a charge carrier at energy ϵ_k whose form depends on the scattering mechanism, $\vec{v}(\epsilon_k)$ the group velocity at $\vec{k}$ and $f(\epsilon_k) = 1/\left(\exp[(\epsilon_k - \mu)/k_B T] + 1\right)$. Following standard steps (*53*), we integrate over the Brillouin zone to find the total current density $\vec{j}$ and read off the conductivity σ from $j = \sigma E$ to obtain the semiclassical conductivity as before (Eq. S50). We separate the integral into two parts ranging from $-\infty$ to 0 and 0 to ∞ and refer to these as σ_h and σ_e respectively. The total conductivity σ is then given by the sum $\sigma_h + \sigma_e$. The momentum relaxation time for each carrier species is obtained by manipulating $\sigma_{e/h}$ into the standard Drude form (Eq. S51) and reading off $\tau_{e/h}$. Here,

$$n_{e/h} = \frac{2m^*}{\pi\hbar^2} \int_{\Delta/2}^{\infty} d\epsilon \sqrt{\epsilon^2 - \left(\frac{\Delta}{2}\right)^2} \left(-\frac{\partial f_{e/h}(\epsilon)}{\partial \epsilon}\right), \quad \text{(S47)}$$

with $f_{e/h}(\epsilon) = 1/\left(\exp[(\epsilon \mp \mu)/k_B T] + 1\right)$. Following this procedure, we obtain

$$\tau_{e/h}(\Delta) = \frac{\int_{\Delta/2}^{\infty} d\epsilon \frac{\epsilon^2 - (\Delta/2)^2}{|\epsilon|} \tau(\pm\epsilon) \left(-\frac{\partial f_{e/h}(\epsilon)}{\partial \epsilon}\right)}{\int_{\Delta/2}^{\infty} d\epsilon \sqrt{\epsilon^2 - (\Delta/2)^2} \left(-\frac{\partial f_{e/h}(\epsilon)}{\partial \epsilon}\right)}, \quad \text{(S48)}$$

where the '+' and '$-$' are for electrons and holes respecively. The collision times $\tau_{e/h}(\Delta)$ for each carrier species is obtained by substituting $\tau(\pm\epsilon)$ (i.e. the transport scattering time for a quasiparticle at a particular energy ϵ) into Eq. (S48). Note that for electron-hole scattering,

Eq. (S48) is only valid at the charge neutrality point (CNP) $\mu = 0$ as that is the only density at which the relaxation time approximation is valid for total-momentum-conserving electron-hole scattering. The average electron-hole collision time away from the CNP is obtained using the conservation of momentum condition $\tau_{e/h} = (n_e + n_h)/(n_{h/e}) \times \tau_0$.

The calculation of $\tau_{e/h}$ according to Eq. (S48) may be carried out exactly for all scattering mechanisms without difficulty in the zero-gap $\Delta = 0$ situation for all scattering mechanisms we consider. These calculations lead to the simple expressions for $\tau_{e/h}$ of the forms detailed in the main text. Evaluating Eq. (S48) at finite gap $\Delta \neq 0$ is difficult since evaluating $\tau(\pm\epsilon)$ with a gap followed by the energy integral in Eq. (S48) is very demanding computationally. To circumvent this, we assume that for all scattering mechanisms, the expression obtained for the collision time at zero gap, denoted henceforth by $\tau_{e/h}$, may be used as a transport scattering time in place of $\tau(\epsilon_k)$ in Eq. (S46) in the presence of a gap. Physically, this corresponds to assuming that $\tau(\epsilon)$ for electron-phonon, electron-impurity, and electron-hole scattering are determined primarily by the phonon population, impurity concentration and overall phase space availability, all of which are unaffected by the introduction of a gap. The above procedure then yields for the gapped collision time

$$\tau_{e/h}(\Delta) = \tau_{e/h} \times \frac{\int_{\Delta/2}^{\infty} d\epsilon \frac{\epsilon^2 - (\Delta/2)^2}{|\epsilon|} \left(-\frac{\partial f_{e/h}(\epsilon)}{\partial \epsilon} \right)}{\int_{\Delta/2}^{\infty} d\epsilon \sqrt{\epsilon^2 - (\Delta/2)^2} \left(-\frac{\partial f_{e/h}(\epsilon)}{\partial \epsilon} \right)}. \tag{S49}$$

In this manner, we are able to evaluate collision times $\tau_{e/h}(\Delta)$ numerically at arbitrary gap Δ given their values at $\Delta = 0$. The validity of the above procedure is validated by the good agreement with experiment demonstrated in the main text. We remind that for electron-hole scattering, Eq. (S49) may only be applied to the electron-hole scattering time τ_0 at CNP. The electron-hole scattering time away from CNP is then obtained by $\tau_{e/h} = (n_e + n_h)/(n_{h/e}) \times \tau_0$, where $n_{e/h}$ are given by Eq. (S47).

The calculation for the gapped case $\Delta = 51$ meV is performed as follows. For electron-

impurity and electron-acoustic-phonon scattering, we insert the gapless momentum relaxation time τ as given by the fit parameters in Table 2 into Eq. S49 to find $\tau_{e/h}(\Delta)$. This is the generalisation of Eq. (S19) to the case of finite gap. Physically, this corresponds to assuming that $\tau(\epsilon)$ for electron-phonon, and electron-impurity are determined primarily by the phonon population and impurity concentration respectively. For electron-hole scattering, we use Eq. (S49) at charge neutrality to obtain $\tau_0(\Delta)$. From there, we obtain the electron-hole momentum relaxation time at finite densities using the usual $\tau_{e/h} = (n_e + n_h)/(n_{h/e}) \times \tau_0$, where $n_{e/h}$ are given by Eq. S47. We plot in Fig. S12 the value of $\Delta\sigma/\sigma_{\rm total}$ against chemical potential μ and temperature T for band gaps $\Delta = 0$, 51 meV (see below for details of the calculation for Δ) and impurity densities $n_{\rm imp} = 10^9$, 10^{10}, 10^{11}, 10^{12} cm^{-2}.

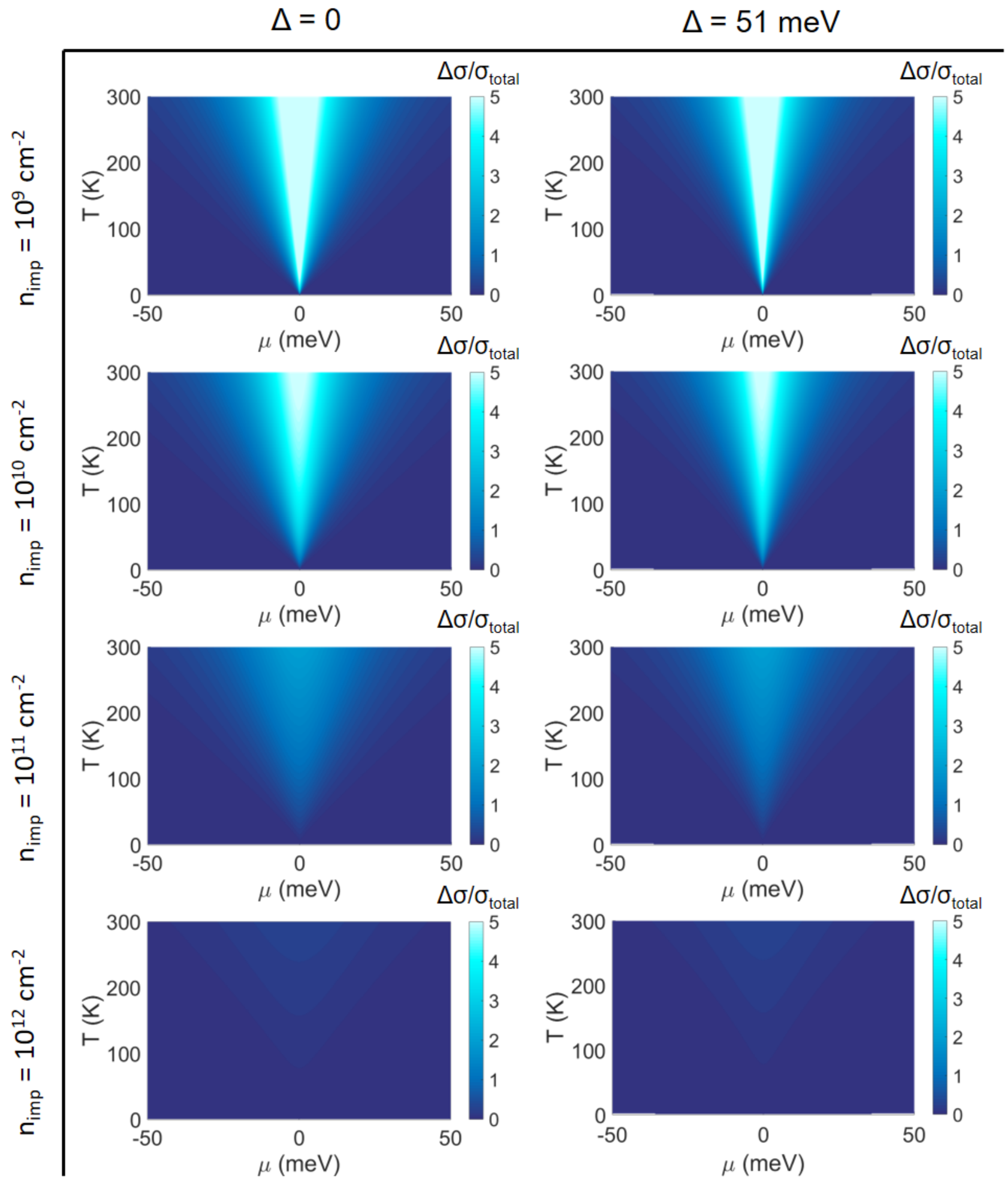

Figure S12: **Hydrodynamic window in a semiconductor.** Normalized reduction in conductivity due to electron-hole scattering $\Delta\sigma/\sigma_{\mathrm{total}}$ as a function of chemical potential μ and temperature T for Δ = 0 and 51 meV at various impurity densities n_{imp}. Increasing impurity density shrinks and eventually closes the window for hydrodynamic conductivity.

6 Evidence of universality

The term "universal" is used in different contexts to mean different things. The purpose of this section is to define how our hydrodynamic transistor is universal. To situate this issue in a broader context, we note that in some communities the term universal is reserved for phenomena that are metrologically precise – for example, the quantum Hall effect is now used for the SI definition of the Ohm (*54*). The driving force for this switch was after the observation of the quantum Hall effect in monolayer graphene, after which it was understood that the phenomena remained universal regardless of whether the material had linear or quadratic bands. However, in the mesoscopic community, phenomena is considered universal when it becomes independent of impurity concentration. Since the concentration of defects varies from sample-to-sample, in this context, universal implies that the phenomena would not exhibit sample-to-sample fluctuations. For example, "universal conductance fluctuations" (UCF) (*17*) are considered universal because the variance of the conductance is independent of impurity concentration (provided the temperature is sufficiently low that phase-coherence length is larger than the sample-size). In practice, the magnitude of UCF does depend on factors like the degree of spin-orbit coupling and the applied magnetic field, and since many experiments are done at temperatures where the phase-coherence length is smaller than the sample size, the observed UCF depends on material parameters like the effective mass and impurity concentrations through the phase-coherence length (*55*). Another example is the minimum conductivity of graphene. The first experimental transport measurements showed remarkable insensitivity to disorder (*56*), and for this reason many at the time believed that a universal mechanism was responsible. However, we showed that this apparent universality arose from a delicate cancellation between carriers induced by impurities and the scattering of these carriers off these impurities (*48*). We predicted that the mechanism was not universal, and that there would be a logarithmic increase of the minimum

conductivity with decreasing disorder, and effect largely confirmed experimentally (*49*).

It is in this context that the hydrodynamic conductivity should be understood. As the impurity concentration is further reduced such that electron-hole scattering becomes the dominant scattering mechanism, there emerges two unconventional contributions to the hydrodynamic conductivity. The first contribution depends on extrinsic electron scattering mechanisms such as charged impurities or acoustic phonons. However, it is not the usual scattering of electrons (or holes) off impurities, but the collective scattering of the electron-hole plasma. Far from neutrality it reduces to the usual Drude diffusive transport. This dissipative contribution, while unusual, is not universal. It dominates away from charge neutrality and depends sensitively on both the choice of platform (bilayer graphene in our case) as well as impurity concentration. For the second contribution, however, all the extrinsic factors, such as impurity concentration and electron-phonon coupling constant drop out, and we also demonstrate that the $\sigma = (e^2/h)(8\log 2/\alpha_0)$ at charge neutrality. As in the UCF example, that independence of the phenomena on impurity concentration (or sample-to-sample variation), is an example of a universal phenomena. However, the universality in our work is even stronger since in addition to no sample-to-sample variation, material parameters like effective mass also drop out. In Figure S13, we show the inverse lifetime by temperature, i.e. $\hbar/(\tau k_B T)$ from G_0W-RPA calculation for gapless parabolic bands as a function of $m^\star e^4/(\hbar^2\kappa^2 k_B T)$. In the limit of sufficiently strong Coulomb interaction and large effective mass, i.e $m^\star e^4/(\hbar^2\kappa^2 k_B * T) \gg 1$, $\hbar/\langle\tau\rangle = 0.35 k_\mathrm{B} T$. This is a stronger example of universal in which the phenomena is independent of material parameters, and it is common in the literature to call such phenomena universal (e.g. Planckian resistivity (*57*)) once it becomes independent of effective mass.

6.1 Theoretical evidence of universality: generalization to all strongly interacting hydrodynamic materials with a hyperbolic dispersion

For elastic processes such as electron-impurity and electron-acoustic phonon scattering, the collision operator in the Boltzmann equation may be manipulated to a "relaxation time form" i.e. $-(f(\epsilon)-f_0(\epsilon))/\tau(\epsilon)$, where $f(\epsilon)$ and $f_0(\epsilon)$ are the non-equilibrium and equilibrium distribution functions respectively and $\tau(\epsilon)$ is the transport scattering time at energy ϵ. Then, one can use standard methods (*35*) to obtain the conductivity in the form of

$$\sigma = e^2 \int_{-\infty}^{\infty} d\epsilon D(\epsilon) \frac{(v_F(\epsilon))^2 \tau(\epsilon)}{2} \left(-\frac{\partial f(\epsilon)}{\partial \epsilon} \right), \tag{S50}$$

which can then be expressed in the Drude form

$$\sigma_{e/h} = \frac{n_{e/h} e^2 \tau_{\mathrm{e/h}}}{m^*}, \tag{S51}$$

giving the energy-averaged transport scattering time as shown in Eq. (S19). However, for inelastic processes such as electron-hole scattering, in general, this procedure is not possible. Instead, a reasonable way to define the momentum relaxation time would be to first calculate the conductivity σ by rigorously solving the Boltzmann equation containing the electron-hole collision operator, then substitute this into the Drude expression $\sigma = ne^2\tau/m^*$ and read off τ. For monolayer graphene, this has been done numerically using a convergent set of basis functions (see e.g. Ref. (*58*) and references therein), but to our knowledge, the analogous calculation for bilayer graphene has not yet been done. For monolayer graphene, the conductivity is log-divergent introducing a non-universal scale t/T (where t is the intralayer hopping) and so the numerical value of the conductivity at a given temperature is not so interesting. For bilayer graphene, as we show in the main text, there is no such log-divergence, the conductivity is universal and temperature independent. Moreover, in what follows we argue that energy-averaged electron-hole momentum relaxation time $\tau_0 = \hbar/(\alpha_0 k_B T)$ with $\alpha_0 = 0.225 \pm 0.002$ extracted

from experiment should be universally observable in all strongly interacting ambipolar hydrodynamic materials with the same dispersion.

In the absence of a fully convergent numerical solution of the quantum Boltzmann equation, a common approach is to expand the non-equilbrium distribution function using a finite number of modes. This was done for bilayer graphene by Ref. (*11*) using the Thomas-Fermi approximation and in Ref. (*12*) using the leading order temperature expansion for the polarizability. These two contemporaneous studies found $\alpha_0 = 0.15$ and 0.29, respectively. In our approach, we use another common method which is to approximate the energy-averaged electron-hole transport scattering time using the energy-averaged electron-hole quasiparticle lifetime which can then be obtained using the full Random Phase Approximation (RPA). This is equivalent to assuming a relaxation time of the form $-(f(\epsilon) - f_0(\epsilon))/\tau(\epsilon)$ for the electron-hole collision operator in the Boltzmann equation, and substituting the electron-hole quasiparticle lifetime in place of $\tau(\epsilon)$ as an effective transport scattering time. In Fig. S13 we show the inverse quasiparticle lifetime calculated using the finite-temperature RPA polarizability. As expected, for sufficiently large Coulomb interactions $m(e^2/\kappa)^2/T \gg 1$, our numerics show that it quickly saturates to a constant universal value 0.356, and that bilayer graphene (black dot) is already approaching this limit. All of these different (and contemporaneous) estimates for α_0 agree to within a factor of ~ 2. An intermediate approach is to map the electron-hole collision operator onto a "relaxation time form" using a generalization of the Bhatnagar-Gross-Krook formalism, where the quasiparticle lifetime is always smaller than the transport scattering time and found (numerically) that they differ by at most a factor of ~ 3. Given this history, we find it reasonable to use the energy-averaged electron-hole quasiparticle lifetime (see Sec. 3.4) within a full RPA approximation to estimate the energy-averaged transport scattering time.

We note that at charge neutrality and zero gap, there exist only two energy scales in the system – temperature $k_B T$ and the Coulomb energy $m^* e^4/\hbar^2\kappa^2$. Therefore, $1/\langle\tau_0\rangle$ must be a func-

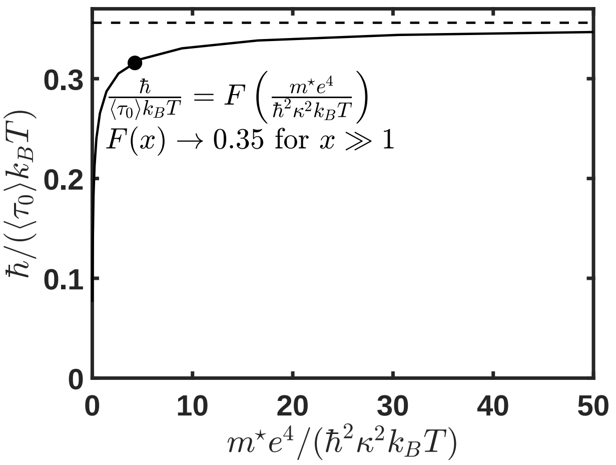

Figure S13: **Universal limit for the electron-hole lifetime.** The inverse energy-averaged electron-hole quasiparticle lifetime for gapless parabolic bands $\epsilon_\pm = \pm\hbar^2k^2/2m^*$ tends to a universal form $1/\langle\tau_0\rangle = 0.35k_BT/\hbar$ in the limit of strong Coulomb interaction and large effective mass $m^*e^4/(\hbar^2\kappa^2) \gg k_BT$. The black circle denotes the bilayer graphene with $\kappa = 3.5$, $T = 200$ K and $m^* = 0.033m_e$.

tion of only these two energy scales. (Here, $\langle\tau_0\rangle$ refers to Eq. (S24) evaluated at charge neutrality and energy-averaged using Eq. (S19). Within the RPA, we find that $\hbar/(k_BT\langle\tau_0\rangle)$ is a function only of the ratio of these two energy scales $m^*e^4/(\hbar^2\kappa^2k_BT)$, and becomes universal when the ratio is large. Using the same argument, we believe that the energy-averaged transport scattering time also shows the same universal behavior as the quasiparticle lifetime. Moreover, we can make the same argument using dimensional analysis. Since the charge-neutral conductivity σ_0 is temperature-independent, it is therefore an experimental fact that the energy-averaged transport scattering time τ_0 goes as $1/T$ since density increases linearly in T. The only functional dependence of τ_0 consistent with $1/T$-linearity is $1/\tau_0 = F\left(m^*e^4/\hbar^2\kappa^2k_BT\right)k_BT/\hbar$, where $F(x)$ is a dimensionless function that can depend on x only through x^0. In other words, F cannot depend on system-specific parameters such as m^* and κ. This convinces us that our experimentally observed value of $\alpha_0 = 0.225 \pm 0.002$ is universal.

Having established that the quasiparticle lifetime is a good estimate of the transport scat-

tering time, we substitute $\tau(\epsilon_{k,\gamma})$ in Eq. (S24) into Eq. (S19) at charge neutrality and zero gap and evaluate the resulting expression over a range of temperatures to read off the prefactor $\alpha_{0,qp}$ in $\tau_{0,qp}^{-1} = \alpha_{0,qp} k_B T/\hbar$. This is to be compared against the actual α_0 in the energy-averaged transport scattering time τ_0, for which $\tau_0^{-1} = \alpha_0 k_B T/\hbar$. We find that $\alpha_{0,qp} = 0.32$ for bilayer graphene at 200 K. We note that the $\alpha_{0,qp}$ obtained from quasiparticle lifetime is 1.5 times *larger* than that obtained from our experiment ($\alpha_0 = 0.225$), as expected since transport scattering time is always larger than lifetime as mentioned above. In the limit of strong Coulomb interaction $m^* e^4/(\kappa^2\hbar^2) \gg k_B T$ and large effective mass $m^\star$, the value of $\tau_{0,qp}^{-1}$ is independent of material-specific parameters m^* and κ, making the value of $\alpha_{0,qp} = 0.356$ in $\tau_{0,qp}^{-1}$ universally applicable in all strongly interacting materials with the same dispersion as bilayer graphene. Consistent with our expectations, the value for α_0 resulting from transport scattering time is slightly smaller than that from the quasiparticle lifetime as explained earlier. We therefore expect that the universal conductivity demonstrated in this work will be reproducible in all strongly interacting ambipolar hydrodynamic materials with the same dispersion.

6.1.1 Analytic calculation of α_0

Substituting $\tau(\epsilon)$ in Eq. (S24) into the energy average formula in Eq. (S19), we numerically evaluate the average scattering rate $\langle\tau\rangle_0$ at charge neutrality and find that it is given by $0.356 k_B T/\hbar$ as shown in Fig. S13. We can make some approximations to obtain analytical results that shed light on how this limit emerges. In particular, Eq. (S24) is the sum of a quasi-electron term,

$$\begin{aligned} \frac{1}{\tau_{\mathrm{qe}}^{(eh)}(\epsilon_\lambda(\vec{k}))} &= \frac{2\pi g}{\hbar}\int\frac{d^2q}{(2\pi)^2}\int\frac{d^2k'}{(2\pi)^2}\sum_{\lambda',\lambda"}|W_{\lambda,\lambda"}|^2 \\ &\quad \times\left\{n_{\vec{k}',-\lambda}\left(1-n_{\vec{k}'+\vec{q},\lambda'}\right)\left(1-n_{\vec{k}-\vec{q},\lambda"}\right)\right\}F^{-\lambda,\lambda'}_{\vec{k}',\vec{k}'+\vec{q}}F^{\lambda,\lambda"}_{\vec{k},\vec{k}-\vec{q}} \\ &\quad \delta\left(\epsilon_{\vec{k}-\vec{q},\lambda"}+\epsilon_{\vec{k}'+\vec{q},\lambda'}-\epsilon_{\vec{k},\lambda}-\epsilon_{\vec{k}',-\lambda}\right) \end{aligned} \quad \text{(S52)}$$

and a quasi-hole term that is given by the above equation with all the Fermi distribution functions replaced by one minus themselves (i.e. the probability of an electron being present is replaced by the probability of a hole being present). The quantity $|W_{\lambda,\lambda"}|^2$ is the screened Coulomb interaction,

$$W_{\lambda,\lambda"} = \frac{\frac{2\pi e^2}{\kappa q}}{1 - \chi^{(0)}(q,\omega)\frac{2\pi e^2}{\kappa q}}.$$

We simplify Eq. (S52) in the low energy regime $\epsilon_\lambda(\vec{k}) \to 0$ and show that linear-in-T behavior results. Because the most important contributions to Eq. (S52) are expected to come from the $q \to 0$ regime, we approximate the screened Coulomb interaction $W_{\lambda,\lambda"} = -\left[\chi^{(0)}(0,0)\right]^{-1}$, and approximate $\chi^{(0)}(0,0)$ as the density of states $gm^*/2\pi\hbar^2$. The two integrals are cut off by temperature since scattering can only take place within a window of size k_BT due to Pauli-blocking. The upper limits of the radial integrals are thus defined by $\hbar^2 q^2/2m^* = k_BT$ and $\hbar^2 k'^2/2m^* = k_BT$ and the Fermi functions are approximated as equal to $1/2$ within this range of integration. The chirality factors are set to unity as they are not expected to cause any difference to the order of magnitude of the final result. Finally, we consider $\lambda = 1$, and neglect interband transitions due to their small contribution compared to intraband. This means that $\lambda'' = 1$ and $\lambda' = -1$. Under these assumptions, Eq. (S52) becomes

$$\frac{1}{\tau_{\text{qe}}^{(eh)}(0)} = \frac{k_BT}{\hbar}\frac{1}{4\pi g}\int_0^1 d\tilde{q}\int_0^{2\pi} d\theta_{\tilde{q}}\int_0^1 d\tilde{k}'\int_0^{\pi} d\theta_{\tilde{k}'}\delta\left(\cos(\theta_{\tilde{k}'})\right),$$

where we have defined $\tilde{k'}^2 = \hbar^2 k'^2/2m^* k_BT$, $\tilde{q}^2 = \hbar^2 q^2/2m^* k_BT$. Working out the integrals yields $1/\tau_{\text{qe}}^{(eh)}(0) = (1/8)k_BT/\hbar$. It can be verified that the quasi-hole term contribution is equal to the quasi-electron term, as it must by electron-hole symmetry. Adding together the quasi-electron and quasi-hole terms in Eq. (S24), we obtain the result that $1/\tau^{(eh)}(0) = (1/4)k_BT/\hbar$. This analytical result of dissipation at $1/4$ is very close to our full numerical solution of 0.356, which is reasonable because the energy average in Eq. (S19) only involves energies close to zero.

6.2 Experimental evidence of universality: Conductivity collapse curve at $\mu = 0$

Here we derive the collapse curve in Fig. 4(C) of the main text. Upon substituting the appropriate expressions for density and electron-hole momentum relaxation time $\tau_{e/h}(\Delta)$ into Eq. (1) of the main text, the electron-hole limited conductivity at charge neutrality $\sigma_{eh}(\Delta)$ normalized by its gapless value $\sigma_{eh}(\Delta = 0)$ collapses as a function of the ratio $k_B T/\Delta$ according to the fit parameter-free function

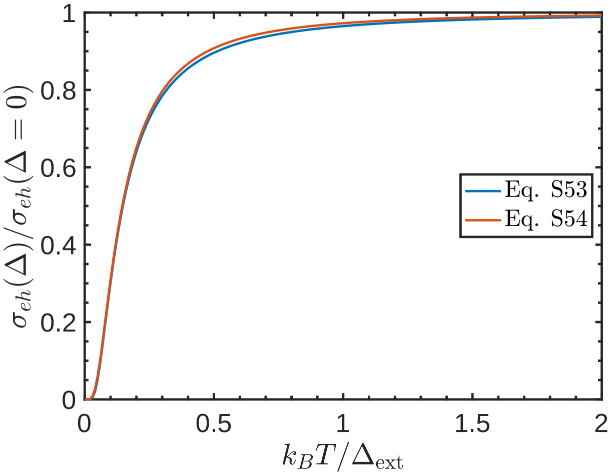

Figure S14: **Validation of Equation S54.** Comparison of the collapse curves of conductivity at charge neutrality against $k_B T/\Delta_{\rm ext}$ shown in Eqs. S53 (numerical) and S54 (analytic). The two curves are in excellent agreement.

$$\frac{\sigma_{eh}(\Delta)}{\sigma_{eh}(\Delta=0)} = 1 + \frac{1}{\log(2)}\left[\log\left(\cosh\left(\frac{\Delta}{4k_BT}\right)\right) - \frac{\Delta}{4k_BT}\tanh\left(\frac{\Delta}{4k_BT}\right) - \left(\frac{\Delta}{2k_BT}\right)^2 \int_{\Delta/2k_BT}^{\infty} dx \frac{1}{4|x|}\frac{1}{\cosh^2(x/2)}\right]. \tag{S53}$$

While the integral in the final term has no exact analytical solution, it is trivial to work out numerically and may be excellently approximated using a function of the form $F(x) = Ax^2\exp(-Bx)$,

where $x \equiv \Delta/k_BT$ and A, B are numerical fit parameters. We find that setting $A = 1/8$ and $B = 5/8$ reproduces the exact expression to an extent almost indistinguishable to the eye. Making use of this approximation for the final term in Eq. (S53), we obtain

$$\frac{\sigma_{eh}(\Delta)}{\sigma_{eh}(\Delta = 0)} = 1 + \tfrac{1}{\log(2)} \left[\log\left(\cosh\left(\tfrac{\Delta}{4k_BT}\right)\right) - \tfrac{\Delta}{4k_BT}\tanh\left(\tfrac{\Delta}{4k_BT}\right) - \tfrac{1}{8}\left(\tfrac{\Delta}{k_BT}\right)^2 \exp\left(-\tfrac{5\Delta}{8k_BT}\right)\right], \quad \text{(S54)}$$

which is Eq. (2) of the main text. We stress that there are no fits to experimental data here and the parameters A and B are introduced only to remove the inconvenience of the numerical integral in Eq. (S53). We show in Fig. S14 that the curves produced by equations (S53) and (S54) are almost identical. Here we have used the same linear relationship $\Delta_{\text{ext}} \approx 2.6\Delta$ as Fig. 4(A) of the main text.

We note that a similar collapse occurs for the commonly encountered case of gapped parabolic bands $\epsilon_\pm(k) = \pm\left(\Delta/2 + \hbar^2k^2/(2m^*)\right)$. In this case, following the same steps outlined above, the same collapse obtains but with the last term in square brackets removed. That is,

$$\frac{\sigma_{eh}^{(para)}(\Delta)}{\sigma_{eh}^{(para)}(\Delta = 0)} = 1 + \frac{1}{\log(2)} \left[\log\left(\cosh\left(\frac{\Delta}{4k_BT}\right)\right) - \frac{\Delta}{4k_BT}\tanh\left(\frac{\Delta}{4k_BT}\right)\right]. \quad \text{(S55)}$$

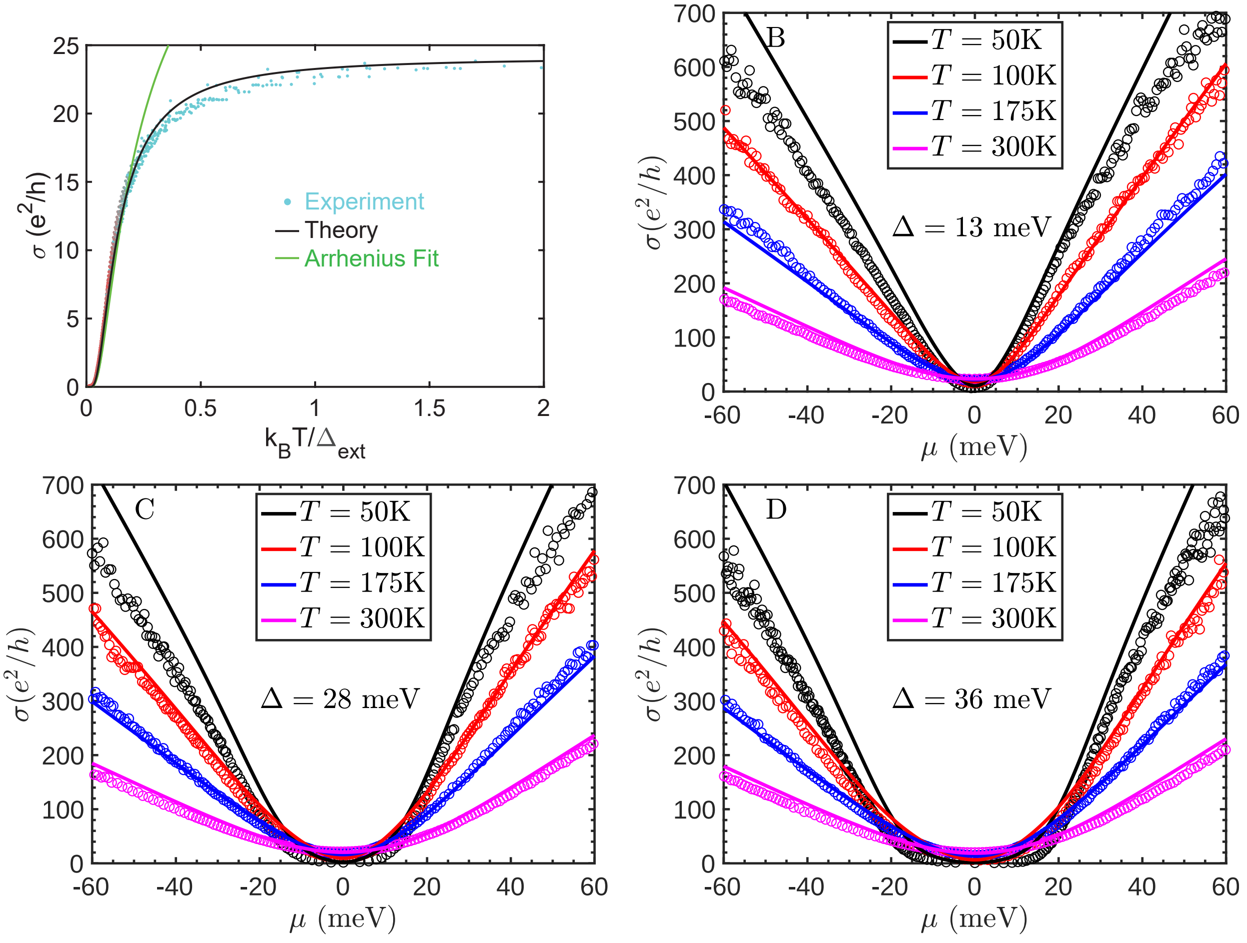

Figure S15: **Additional data for the gapped conductivity.** (A) Renormalized charge neutral conductivity plotted against k_BT/Δ_{ext}. The data is observed to collapse in agreement with ambipolar hydrodynamic conductivity in the strong electron-hole scattering regime, and in disagreement with the Arrhenius fit. (B-D) Comparison of theory (solid lines) in the strong electron-hole scattering limit against experimental data (circles) at four different temperatures and three different band gaps. The same single set of fit parameters is used throughout.

6.3 Further comparisons of theory and experiment at different gaps

We present in Fig. S15 the collapse of the charge neutral conductivity as a function of k_BT/Δ_{ext} up to room temperature without normalization. Here we have $\alpha_0 = 0.225$ as obtained by fitting to σ_0 in the main text. Note that all the other scattering parameters $\alpha_{ac}^{e/h}$, and τ_i play no role

at charge neutrality regardless of the gap. As seen in the figure, theory collapses in excellent agreement with experiment. We note that this collapse deviates from Arrhenius behavior, as shown from fitting the experimental results to the Arrhenius equation $\sigma = A \exp(-\Delta/2k_B T)$, where A is a numerical fit parameter and $\Delta = \Delta_{\text{ext}}/2.6$. As stated in the text, we compare theory against experiment at different gaps in Fig. S15 below, using the same single set of four fit parameters featured in Table 2 in Eqs. (S12) and (S13). At all gaps considered, experiment agrees with theory using only the fit parameters extracted at zero gap.

REFERENCES AND NOTES

1. R. N. Gurzhi, Hydrodynamic effects in solids at low temperature. *Sov. Phys. Uspekhi* **11**, 255–270 (1968).

2. B. N. Narozhny, I. V. Gornyi, M. Titov, M. Schütt, A. D. Mirlin, Hydrodynamics in graphene: Linear-response transport. *Phys. Rev. B* **91**, 035414 (2015).

3. M. Dyakonov, Shallow water analogy for a ballistic field effect transistor: New mechanism of plasma wave generation by dc current. *Phys. Rev. Lett.* **71**, 2465–2468 (1993).

4. H. Predel, H. Buhmann, L. W. Molenkamp, R. N. Gurzhi, A. N. Kalinenko, A. I. Kopeliovich, A. V. Yanovsky, Effects of electron-electron scattering on electron-beam propagation in a two-dimensional electron gas. *Phys. Rev. B* **62**, 2057–2064 (2000).

5. A. O. Govorov, J. J. Heremans, Hydrodynamic effects in interacting fermi electron jets. *Phys. Rev. Lett.* **92**, 026803 (2004).

6. M. Müller, L. Fritz, S. Sachdev, Quantum-critical relativistic magnetotransport in graphene. *Phys. Rev. B* **78**, 115406 (2008).

7. M. Müller, J. Schmalian, L. Fritz, Graphene: A nearly perfect fluid. *Phys. Rev. Lett.* **103**, 025301 (2009).

8. C. B. Mendl, M. Polini, A. Lucas, Coherent terahertz radiation from a nonlinear oscillator of viscous electrons. *Appl. Phys. Lett.* **118**, 013105 (2021).

9. J. Zaanen, Electrons go with the flow in exotic material systems. *Science* **351**, 1026–1027 (2016).

10. D. Y. H. Ho, I. Yudhistira, N. Chakraborty, S. Adam, Theoretical determination of hydrodynamic window in monolayer and bilayer graphene from scattering rates. *Phys. Rev. B* **97**, 121404 (2018).

11. M. Zarenia, T. B. Smith, A. Principi, G. Vignale, Breakdown of the Wiedemann-Franz law in *AB*-stacked bilayer graphene. *Phys. Rev. B* **99**, 161407 (2019).

12. G. Wagner, D. X. Nguyen, S. H. Simon, Transport in bilayer graphene near charge neutrality: Which scattering mechanisms are important? *Phys. Rev. Lett.* **124**, 026601 (2020).

13. P. Gallagher, C.-S. Yang, T. Lyu, F. Tian, R. Kou, H. Zhang, K. Watanabe, T. Taniguchi, F. Wang, Quantum-critical conductivity of the Dirac fluid in graphene. *Science* **364**, 158–162 (2019).

14. Y. Nam, D.-K. Ki, D. Soler-Delgado, A. F. Morpurgo, Electron-hole collision limited transport in charge-neutral bilayer graphene. *Nat. Phys.* **13**, 1207–1214 (2017).

15. D. X. Nguyen, G. Wagner, S. H. Simon, Quantum boltzmann equation for bilayer graphene. *Phys. Rev. B* **101**, 035117 (2020).

16. C. A. Kukkonen, P. F. Maldague, Electron-hole scattering and the electrical resistivity of the semimetal TiS_2. *Phys. Rev. Lett.* **37**, 782–785 (1976).

17. P. A. Lee, A. D. Stone, Universal conductance fluctuations in metals. *Phys. Rev. Lett.* **55**, 1622–1625 (1985).

18. D. A. Bandurin, I. Torre, R. K. Kumar, M. B. Shalom, A. Tomadin, A. Principi, G. H. Auton, E. Khestanova, K. S. Novoselov, I. V. Grigorieva, L. A. Ponomarenko, A. K. Geim, M. Polini, Negative local resistance caused by viscous electron backflow in graphene. *Science* **351**, 1055–1058 (2016).

19. J. Crossno, J. K. Shi, K. Wang, X. Liu, A. Harzheim, A. Lucas, S. Sachdev, P. Kim, T. Taniguchi, K. Watanabe, T. A. Ohki, K. C. Fong, Observation of the Dirac fluid and the breakdown of the Wiedemann-Franz law in graphene. *Science* **351**, 1058–1061 (2016).

20. A. I. Berdyugin, S. G. Xu, F. M. D. Pellegrino, R. K. Kumar, A. Principi, I. Torre, M. B. Shalom, T. Taniguchi, K. Watanabe, I. V. Grigorieva, M. Polini, A. K. Geim, D. A. Bandurin, Measuring Hall viscosity of graphene's electron fluid. *Science* **364**, 162–165 (2019).

21. C. R. Dean, A. F. Young, I. Meric, C. Lee, L. Wang, S. Sorgenfrei, K. Watanabe, T. Taniguchi, P. Kim, K. L. Shepard, J. Hone, Boron nitride substrates for high-quality graphene electronics. *Nat. Nanotechnol.* **5**, 722–726 (2010).

22. J. K. Viljas, T. T. Heikkilä, Electron-phonon heat transfer in monolayer and bilayer graphene. *Phys. Rev. B* **81**, 245404 (2010).

23. X. Li, K. M. Borysenko, M. B. Nardelli, K. W. Kim, Electron transport properties of bilayer graphene. *Phys. Rev. B* **84**, 195453 (2011).

24. M. Lv, S. Wan, Screening-induced transport at finite temperature in bilayer graphene. *Phys. Rev. B* **81**, 195409 (2010).

25. M. Kohler, Allgemeine theorie der abweichungen von der mathiessenschen regel. *Z. Phys.* **126**, 495–506 (1949).

26. J. Zaanen, Why the temperature is high. *Nature* **430**, 512–513 (2004).

27. K. Zou, X. Hong, J. Zhu, Effective mass of electrons and holes in bilayer graphene: Electron-hole asymmetry and electron-electron interaction. *Phys. Rev. B* **84**, 085408 (2011).

28. T. V. Phan, J. C. W. Song, L. S. Levitov, Ballistic Heat Transfer and Energy Waves in an Electron System. arXiv:1306.4972 (2013).

29. R. Takahashi, M. Matsuo, M. Ono, K. Harii, H. Chudo, S. Okayasu, J. Ieda, S. Takahashi, S. Maekawa, E. Saitoh, Spin hydrodynamic generation. *Nat. Phys.* **12**, 52–56 (2016).

30. L. Wang, I. Meric, P. Y. Huang, Q. Gao, Y. Gao, H. Tran, T. Taniguchi, K. Watanabe, L. M. Campos, D. A. Muller, J. Guo, P. Kim, J. Hone, K. L. Shepard, C. R. Dean, One-dimensional electrical contact to a two-dimensional material. *Science* **342**, 614–617 (2013).

31. E. McCann, Asymmetry gap in the electronic band structure of bilayer graphene. *Phys. Rev. B* **74**, 161403 (2006).

32. K. M. Borysenko, J. T. Mullen, X. Li, Y. G. Semenov, J. M. Zavada, M. B. Nardelli, K. W. Kim, Electron-phonon interactions in bilayer graphene. *Phys. Rev. B* **83**, 161402 (2011).

33. J. Huang, J. A. Alexander-Webber, T. J. B. M. Janssen, A. Tzalenchuk, T. Yager, S. Lara-Avila, S. Kubatkin, R. L. Myers-Ward, V. D. Wheeler, D. K. Gaskill, R. J. Nicholas, Hot carrier relaxation of dirac fermions in bilayer epitaxial graphene. *J. Phys. Condens. Matter* **27**, 164202 (2015).

34. D. K. Efetov, P. Kim, Controlling electron-phonon interactions in graphene at ultrahigh carrier densities. *Phys. Rev. Lett.* **105**, 256805 (2010).

35. S. Das Sarma, S. Adam, E. H. Hwang, E. Rossi, Electronic transport in two-dimensional graphene. *Rev. Mod. Phys.* **83**, 407–470 (2011).

36. P. S. Alekseev, A. P. Dmitriev, I. V. Gornyi, V. Y. Kachorovskii, B. N. Narozhny, M. Schütt, M. Titov, Magnetoresistance of compensated semimetals in confined geometries. *Phys. Rev. B* **95**, 165410 (2017).

37. E. McCann, M. Koshino, The electronic properties of bilayer graphene. *Rep. Prog. Phys.* **76**, 056503 (2013).

38. E. McCann, V. I. Fal'ko, Landau-level degeneracy and quantum Hall effect in a graphite bilayer. *Phys. Rev. Lett.* **96**, 086805 (2006).

39. H. Ochoa, E. V. Castro, M. I. Katsnelson, F. Guinea, Temperature-dependent resistivity in bilayer graphene due to flexural phonons. *Phys. Rev. B* **83**, 235416 (2011).

40. D. Svintsov, V. Vyurkov, S. Yurchenko, T. Otsuji, V. Ryzhii, Hydrodynamic model for electron-hole plasma in graphene. *J. Appl. Phys.* **111**, 083715 (2012).

41. K. Kaasbjerg, K. S. Thygesen, K. W. Jacobsen, Unraveling the acoustic electron-phonon interaction in graphene. *Phys. Rev. B* **85**, 165440 (2012).

42. T. Sohier, M. Calandra, C.-H. Park, N. Bonini, N. Marzari, F. Mauri, Phonon-limited resistivity of graphene by first-principles calculations: Electron-phonon interactions, strain-induced gauge field, and Boltzmann equation. *Phys. Rev. B* **90**, 125414 (2014).

43. M. Combescot, R. Combescot, Conductivity relaxation time due to electron-hole collisions in optically excited semiconductors. *Phys. Rev. B* **35**, 7986–7992 (1987).

44. J. Schiefele, F. Sols, F. Guinea, Temperature dependence of the conductivity of graphene on boron nitride. *Phys. Rev. B* **85**, 195420 (2012).

45. S. Fratini, F. Guinea, Substrate-limited electron dynamics in graphene. *Phys. Rev. B* **77**, 195415 (2008).

46. V. Perebeinos, P. Avouris, Inelastic scattering and current saturation in graphene. *Phys. Rev. B* **81**, 195442 (2010).

47. M. Polini, G. Vignale, V. Pellegrini, J. K. Jain, The quasiparticle lifetime in a doped graphene sheet, in *No-Nonsense Physicist: An Overview of Gabriele Giuliani's Work and Life* (Scuola Normale Superiore, 2016), pp. 107–124.

48. S. Adam, E. H. Hwang, V. M. Galitski, S. D. Sarma, A self-consistent theory for graphene transport. *Proc. Natl. Acad. Sci. U.S.A.* **104**, 18392–18397 (2007).

49. D. Rhodes, S. H. Chae, R. Ribeiro-Palau, J. Hone, Disorder in van der waals heterostructures of 2d materials. *Nat. Mater.* **18**, 541–549 (2019).

50. M. Müller, S. Sachdev, Collective cyclotron motion of the relativistic plasma in graphene. *Phys. Rev. B* **78**, 115419 (2008).

51. X.-F. Wang, T. Chakraborty, Coulomb screening and collective excitations in biased bilayer graphene. *Phys. Rev. B* **81**, 081402 (2010).

52. L. Jiang, Z. Shi, B. Zeng, S. Wang, J.-H. Kang, T. Joshi, C. Jin, L. Ju, J. Kim, T. Lyu, Y.-R. Shen, M. Crommie, H.-J. Gao, F. Wang, Soliton-dependent plasmon reflection at bilayer graphene domain walls. *Nat. Mater.* **15**, 840–844 (2016).

53. N. Ashcroft, N. Mermin, *Solid State Physics* (Saunders College, 1976).

54. K. von Klitzing, The quantized hall effect. *Rev. Mod. Phys.* **58**, 519–531 (1986).

55. B. L. Altshuler, A. G. Aronov, D. E. Khmelnitsky, Effects of electron-electron collisions with small energy transfers on quantum localisation. *J. Phys. C* **15**, 7367–7386 (1982).

56. A. K. Geim, K. S. Novoselov, The rise of graphene. *Nat. Mater.* **6**, 183–191 (2007).

57. J. A. N. Bruin, H. Sakai, R. S. Perry, A. P. Mackenzie, Similarity of scattering rates in metals showing T-linear resistivity. *Science* **339**, 804–807 (2013).

58. H.-Y. Xie, M. S. Foster, Transport coefficients of graphene: Interplay of impurity scattering, Coulomb interaction, and optical phonons. *Phys. Rev. B* **93**, 195103 (2016).
